# MALNC: a new mutant NPM1/IDH2^R140^ and PML-RARA-associated lncRNA with impact on AML cell proliferation, maturation and drug response

**DOI:** 10.1038/s41417-025-00954-0

**Published:** 2025-08-23

**Authors:** Elisabetta Cozzi, Anne Neddermeyer, Xiangfu Zhong, Angelica María Gamboa-Cedeño, Dimitris C. Kanellis, Albin Österroos, My Björklund, Nona Struyf, Kasper Karlsson, Ying Qu, Alma Månsson, Tatjana Pandzic, Sofia Bengtzén, Christer Nilsson, Roland Fiskesund, Panagiotis Baliakas, Tom Erkers, Jiri Bartek, Olli-Pekka Kallioniemi, Hong Qian, Andreas Lennartsson, Sören Lehmann

**Affiliations:** 1 Department of Medicine, Center for Hematology and Regenerative Medicine, Karolinska Institute, Huddinge, Sweden; 2 Department of Medical Sciences, Hematology, Uppsala University, Uppsala, Sweden; 3 Department of Clinical Genetics, Uppsala University Hospital, Uppsala, Sweden; 4 Department Medical Biochemistry and Biophysics, Karolinska Institute, Solna, Sweden; 5 Department of Oncology and Pathology, Karolinska Institute, Solna, Sweden; 6 Danish Cancer Institute, Copenhagen, Denmark

**Keywords:** Leukaemia, Cancer

## Abstract

As the non-coding genome remains poorly characterized in acute myeloid leukemia (AML), we aimed to identify and functionally characterize novel long non-coding RNAs (lncRNAs) relevant to AML biology and treatment. We first identified lncRNAs overexpressed in AML blasts and, among them, discovered a novel transcript, which we named *myeloid and AML-associated intergenic long non-coding RNA* (MALNC). MALNC is overexpressed in AML, particularly in cases with the *PML-RARA* fusion or *IDH2*^*R140*^*/NPM1* co-mutations, and is associated with a distinct gene expression profile. Functional studies showed that *MALNC* knockout impairs AML cell proliferation and colony formation, enhances ATRA-induced differentiation, and sensitizes cells to arsenic trioxide. Transcriptomic analysis revealed that MALNC loss alters the expression of retinoic acid pathway genes, and chromatin binding studies showed that MALNC binds to genes related to the retinoic acid and Rho GTPase pathways. In conclusion, we have identified MALNC as a novel lncRNA that promotes leukemic cell proliferation, counteracts ATRA-induced differentiation, and modulates drug sensitivity in AML.

## Introduction

Acute myeloid leukemia (AML) is an aggressive myeloid malignancy associated with poor outcomes and a significant unmet medical need [1, 2]. To improve patient care, a deeper understanding of AML biology is crucial. While the coding genome has been thoroughly investigated during the last decades, the non-coding genome, such as long non-coding RNAs (lncRNAs), remains poorly characterized in AML. LncRNAs are non-protein-coding RNA transcripts longer than 200 base pairs (bp) [3] that regulate gene expression, post-translational processing, genome stability and chromatin structure formation. The clinical significance of most lncRNAs and their involvement in AML remain elusive [4, 5]. Functionally, lncRNAs can possess both tumor suppressor and oncogenic functions [6]. Aberrant lncRNA expression in AML has been linked to poorer prognosis [7–9], recurrent genetic aberrations [10–13] and drug resistance in patients [14, 15]. Thus, lncRNAs have also been proposed as potential prognostic and predictive biomarkers [10, 16] and have been implicated in biological functions in AML [7, 15–18].

AML is a genetically heterogeneous disease, with distinct subgroups defined by specific genetic aberrations. Acute promyelocytic leukemia (APL) comprises 5–10% of AML cases and is characterized by the presence of a fusion between the transcription factor *promyelocytic leukemia* (*PML*) and the *retinoic acid receptor* α (*RARA*) genes [19, 20]. The resulting *PML-RARA* fusion gene encodes an oncoprotein (PML-RARα), which acts as a transcriptional repressor and causes a differentiation blockage at the promyelocytic stage [21]. APL survival rates have greatly improved since the introduction of all-*trans* retinoic acid (ATRA) and arsenic trioxide (ATO) treatment [21, 22].

Beyond APL-specific alterations, several recurrent genetic mutations have been observed across other AML subtypes. *NPM1* mutations are among the most common, occurring in 25–35% of all AML cases, and are particularly frequent in AML with a normal karyotype, where they are present in 45- 65% of cases [23, 24]. *NPM1* mutations are associated with a favorable prognosis, especially in the absence of *FLT3* mutations [25, 26]. In contrast, *IDH2* mutations, typically occurring at the R140 and R172 loci, have a less well-defined prognostic impact and are not routinely used for AML risk stratification [25, 27].

Previous studies have shown that the lncRNA HOTAIRM1 is associated with the degradation of PML-RARα, regulating autophagy-mediated degradation of the fusion oncoprotein and thus controlling myeloid differentiation [18, 28]. lncRNAs HOXB-AS3 and LONA have been associated with leukemogenic effects in AML with mutant *NPM1* [29, 30]. Such studies suggest that lncRNAs can be involved in promoting leukemia in certain genetic subgroups of AML. Furthermore, while lncRNAs as SNHG14 and CDK6-AS1 have been implicated in drug responses [17, 31], our understanding of the specific role(s) of lncRNAs in drug sensitivity and resistance remains limited.

In this study, we identified and characterized MALNC, a novel lncRNA that is highly expressed in two genetically defined subgroups of AML. Transcriptomic and functional analyzes revealed that MALNC influences leukemic cell proliferation, differentiation, and drug response. Collectively, our findings suggest that MALNC may have important implications for pathophysiology, classification, prognosis, and drug responses in leukemia.

## Materials and methods

### AML patient cohorts

Initial identification of MALNC lncRNAs was performed using an internal discovery cohort comprising five healthy donor NBM samples and seven newly diagnosed AML patient samples (Supplementary Table 1). Validation of MALNC differential expression was subsequently performed within the same study using an independent internal cohort of AML patients and healthy donors, referred to as the Knut and Alice Wallenberg (KAW) cohort (AML, *n* = 103; NBM, *n* = 11). For broader analyzes of MALNC expression and its association with genetic, transcriptomic, clinical, and prognostic features, two additional AML cohorts were analyzed. Firstly, our own ClinSeq-AML cohort (*n* = 325), consisting of consecutively diagnosed, population-based AML cases in Sweden between 1997 and 2014 [32]. Clinical data were collected from the Swedish Adult Acute Leukemia Registry and individual medical records. Transcriptomic and mutational data were obtained through RNA sequencing and targeted DNA panel sequencing, as previously described [32–34]. Secondly, we used the publicly available TCGA-AML cohort (*n* = 151), composed of diagnostic de novo AML cases biobanked in the United States between 2002 and 2009. Clinical and follow-up data were retrieved from the GDC Data Portal (https://portal.gdc.cancer.gov/projects/TCGA-LAML) and were used in correlation analyzes. All samples and associated data from these cohorts were obtained with informed consent and in accordance with the Declaration of Helsinki. Cohort characteristics are summarized in Supplementary Tables 1-2. In this project, the cytogenetic classification of the ClinSeq cohort was based on the updated MRC classification [35], the genetic classification was based on *NPM1*, *FLT3* and *CEBPA* mutational status according to the 2017 NCCN guidelines [36] and for the ELN classification the ELN2017 criteria was used [37] as full data was not available for ELN2022 for the entire cohort.

### AML cell lines

HL60, NB4, OCI-AML3, OCI-AML2, MOLM-13, and Kasumi-1 cell lines used in this study were purchased from the German Collection of Microorganisms and Cell Cultures (DSMZ). K562, KG1 and U937 cells were obtained from the American Type Culture Collection (ATCC, Manassas, VA, USA). AML cell lines HL60, NB4, U937, IMS-M2 and K562 (chronic myeloid leukemia, CML, cell line) were cultured in RPMI-1640 with GlutaMAX and HEPES (Gibco) supplemented with 10% heat-inactivated fetal bovine serum (FBS, Thermo Scientific), while MOLM-13 and Kasumi1 cells were cultured in medium supplemented with 20% FBS. KG1 cells were cultured in IMDM medium with GlutaMAX™ (Gibco) supplemented with 20% FBS and OCI-AML2 and OCI-AML3 cells were cultured in α-MEM (Gibco) supplemented with 20% FBS. All cell lines were mycoplasma-free and grown in a humidified incubator at 37 °C and 5% CO_2_.

### Gene expression analyzes by RT-qPCR

RNA was extracted using the RNeasy Plus Mini Kit (Qiagen) according to the manufacturer’s protocol. RNA was eluted in nuclease-free water and the concentration was measured using Qubit 3.0 (Invitrogen). Next, RNA was reverse transcribed using a High-Capacity cDNA Reverse Transcription Kit with RNase Inhibitor (Applied Biosystems) containing random hexamers. Then, cDNA concentration was measured using Qubit 3.0. Relative expression was measured by real-time quantitative polymerase chain reaction (RT-qPCR, CFX Touch Real-Time PCR Detection Systems, BioRad) using PrimeTime® Gene Expression Master Mix with 55 ng cDNA input. Relative quantitation was determined by the ΔΔCT method [38] using TBP as an endogenous control mRNA. Sequences of RT-qPCR assays are listed in Supplementary Table 3.

### Subcellular fractionation

Subcellular fractionation was performed using the PARIS fractionation kit (Thermo Scientific), followed by DNase removal (TURBO™ DNA-free™ DNase Treatment, Thermo Scientific) according to the manufacturer´s recommendation. After fractionation, RT-qPCR was performed as described before. ACTB and U6 RNA expression were used as cytoplasmic and nuclear controls, respectively.

### CRISPR-Cas9 knockout of MALNC in AML cells

#### Design of gRNA

Guide RNA (gRNA) targeting *MALNC loci* were designed using CRISPOR v4.8 and GPP sgRNA Design tool (GPP Web Portal, Broad Institute) targeting upstream and downstream *loci* of *MALNC* and/or *MALNC TSS* and controlling for off-target effects.

#### Electroporation

HL60 and NB4 cell lines were transfected with CRISPR-Cas9-gRNA ribonucleoprotein (RNP) complexes using the combination of two gRNAs per deletion. Per transfection, 1 × 10^5^ cells were pelleted, washed once in DPBS (w/o Ca2^+^ and Mg2^+^, Thermo Scientific) and resuspended in 5 µl Resuspension buffer R (Thermo Scientific). RNP complexes were prepared by mixing 12pmol of Cas9 protein (TrueCut™ Cas9 Protein v2, Thermo Scientific, SpCas9 from Streptococcus pyogenes: 5’-NGG-3’) with 6pmol of each gRNA in Resuspension buffer R and incubated at RT for 20 minutes. Assembled RNP complexes and resuspended cells were mixed at equal parts and electroporated using 10 µl NEON electroporation tips (1350 V, 35 ms, 1 pulse). The total RNP volume was below 10% of the final transfection volume. One minute after electroporation, the transfected cells were gently transferred to a 24-well plate with warm medium and incubated for 3–4 days at 37 °C.

#### Genomic cleavage assay

An aliquot of transfected cells was used for genomic DNA (gDNA) extraction (QIAamp DNA Mini Kit, Qiagen), followed by PCR amplification (Phusion Flash High-Fidelity PCR Master Mix, Thermo Scientific) of the intended deletion site using 50 ng of gDNA for each PCR reaction. Successful genomic deletions were confirmed by gel electrophoresis and Sanger sequencing (Eurofins Genomics) of purified PCR product (MinElute Gel Extraction Kit, Qiagen). Sanger-sequencing data were analyzed using TIDE analysis (Tracking of Indels by Decomposition).

#### Single-cell clone generation

After confirmation of deletion on the bulk level, the bulk population was single-cell sorted by FACS onto 96-well plates and incubated for 10–12 days. Deletion in each single-cell-derived cell line was investigated as described above and knockout (KO) cells with homozygous or heterozygous deletion of *MALNC* were selected for functional studies. Control cells (WT) were generated by transfection with only Cas9. RT-qPCR assays, deletion validation primer and gRNA sequences are listed in Supplementary Tables 3, 4 and 5.

### CAGE-sequencing data

Cap analysis of gene expression sequencing (CAGE-seq) data was retrieved from the publicly available Functional Annotation of the Mammalian genome (FANTOM) 5 project (RIKEN, Japan, http://fantom.gsc.riken.jp/5/datafiles/latest/basic/). Data were obtained from leukemic cell lines and normal tissue samples from healthy donors. All samples were processed using hCAGE (standard CAGE using 5 µg total RNA) and the activity of transcription start sites (TSS) within each sample is represented by CAGE tag starting sites (CTSS). CTSS signal correlates with transcript expression level. Data were auto-scaled by group (all represented data tracks) and represented using Integrative Genomes Viewer (IGV, v2.8.0, Broad Institute) [39]. CAGE samples used are listed in Supplementary Table 6.

### Expression in normal tissues

CAGE-seq data from 15 normal healthy tissues were obtained from FANTOM5, as described above. Additionally, expression data from normal healthy tissues was obtained by using Illumina bodyMap2 transcriptome data (RPKM, total RNA-seq, 16 different tissues) (*n* = 1, BioProject: RJEB2445) and HPA RNA-seq normal tissues (RPKM, Total RNA-seq, 27 different tissues (n = 2–7, BioProject: PRJEB4337).

### Proliferation analysis

Cell viability was measured using WST-8 tetrazolium salt (Cell Counting Kit 8, Abcam). Cells were seeded as 10,000 cells/well/100 µl on a clear bottom 96-well plate. For readout, WST-8 was added (10 µl/well), cells incubated for 4 h (37 °C and 5% CO2) and then absorbance of formazan dye formed measured at 460 and 660 nm (reference wavelength) using a Synergy HTX plate reader. Cells were incubated for up to 96 h and readout was performed every 24 h.

### Colony-forming unit in culture

Colony formation was studied using MethoCult™ H4034 Optimum (Stem Cell Technologies). Cells were plated in methylcellulose medium in triplicate using 1000 cells/plate. After 15 days of incubation (37 °C and 5% CO^2^) formed colonies were manually quantified.

### Cell differentiation analysis

LncRNA expression levels were determined during an ATRA-induced granulocytic differentiation assay. Cells were cultured in RPMI-1640 supplemented with 10% FBS and treated with either 1–10 µM ATRA (Sigma-Aldrich) or vehicle control (final concentration 0.01% dimethyl sulfoxide, DMSO, Sigma-Aldrich). Differentiation was confirmed by increased cell surface staining of granulocytic marker CD11b. In short, 1 × 10^6^ cells were washed with PBS (400 *g* for 5 min) and fixated with 1% paraformaldehyde (Sigma-Aldrich) during bottom-to-top rotation for 10 min. The cell pellet was washed twice with ice-cold eBioscience™ Flow Cytometry staining buffer (Thermo Scientific), and re-suspended in 100 μl staining buffer. Next, cells were incubated with 0.5 μg/test anti-CD11b-APC monoclonal antibody (ICRF44, Thermo Scientific) for 30 min on ice and in the dark. After three additional washing steps, the cell pellet was re-suspended in 500 μl staining buffer and flow cytometry data was determined using BC CytoFLEX Flow Cytometer (Beckman Colter). The percentage of CD11b expressing cells was quantified using FlowJo™ v10.6.1. RNA was extracted at different time points and MALNC expression levels were determined using RT-qPCR.

### Chromatin isolation by RNA purification (ChIRP) sequencing analysis

ChIRP sequencing (ChIRP-seq) analysis was performed as previously described [40]. Briefly, antisense biotinylated DNA probes were designed against MALNC transcript through the LGC Biosearch Technologies online software (Supplementary Table 7). NEAT1 and LacZ probes were used as positive and negative controls, respectively. Sequencing libraries were prepared from the entire sample volume of ChIRP-DNA using the Smarter ThruPLEX DNA-seq Kit without Covaris fragmentation (cat# R400676, Takara) with Unique dual index Set A-D (cat# R400665/6/7/8, Takara). The library preparation was performed according to the manufacturer’s instructions (guide#112219). The libraries were sequenced on Illumina NovaSeq 6000 SP flowcells as paired-end 150 bp read length, performed by the SNP&SEQ Technology Platform. The MALNC ChIRP-seq data were processed using the nf-core [41] ChipSeq pipeline (version 2.0.0). In detail, FastQC (version 0.11.9) was used for raw reads QC, Trim Galore! (version 0.6.1) or adapter trimming, BWA (version 0.7.17) for mapping and Picard (version 2.23.4) for duplicate marking. MACS (version 2.2.6) was used for peak calling. The ChIRP-seq data were mapped to the human reference genome hg38. Peaks were first called using MACS2 (-p 1e-5), then overlapped between two biological replicates. The MALNC peaks were annotated by R package ChIPseeker. ChIRP sequencing data are deposited in the Gene Expression Omnibus (GEO) repository under the accession number GSE299541.

### Statistical testing and data analysis

Statistical analyzes were performed using the R statistical package (v3.6.2) and GraphPad Prism (10.4.0). Flow cytometry data were analyzed with FlowJo (v10.6.1–v10.8.0). The sample size was determined based on standard scientific practice, prior studies in similar contexts and the expected variability in the data. Detailed information regarding sample size, biological replicates, and statistical tests used for each experiment is provided in the figure legends. Unless otherwise specified, unpaired Student’s *t*-test was used for comparisons between two groups, assuming normality and homogeneity of variance. If the assumption of equal variances was not met, a Welch’s *t* test was applied. For multiple-group comparisons, one-way analysis of variance (ANOVA) was employed to calculate *p* values. For non-normally distributed data, the Kruskal–Wallis test was applied. In cases where categorical data overlap between the groups was assessed, the hypergeometric test was used to determine if the observed overlap was statistically significant, considering the sample size and distribution of categories in both groups. Data are presented as mean ± SEM. Statistical significance was defined as follows: ns, not significant, p * < 0.05, ** < 0.01, *** < 0.001.

More detailed information on experimental procedures can be found in the Supplementary materials and methods.

## Results

### MALNC is a lncRNA with distinct splice variants and elevated expression in AML

**Fig. 1:**
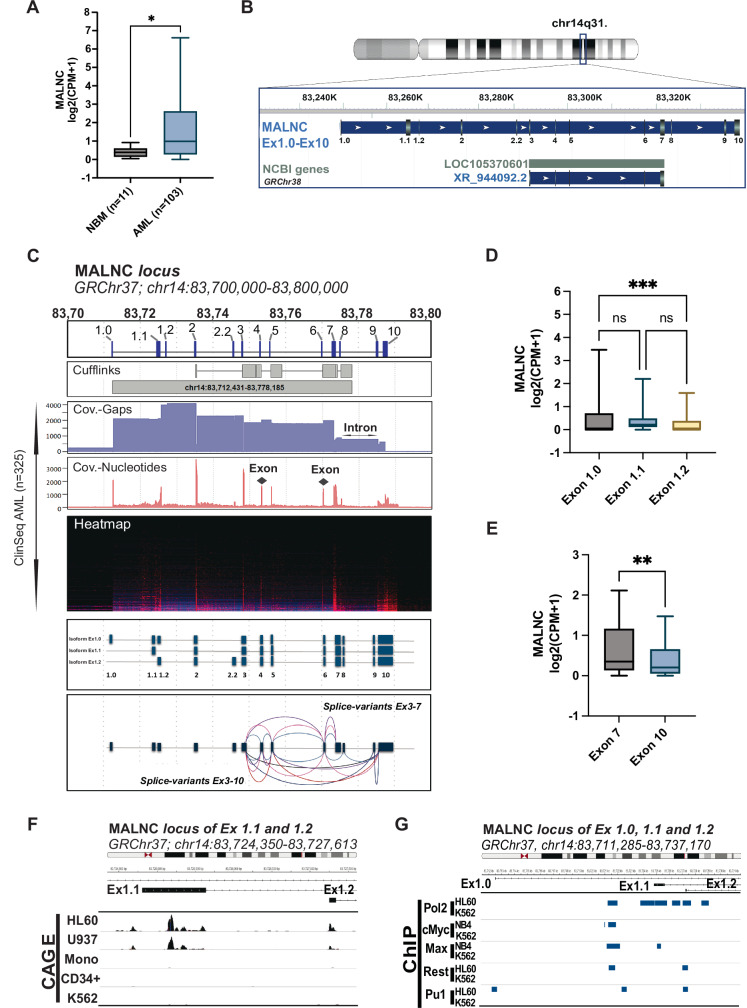
LncRNA MALNC, highly expressed in AML, is multi-exonic and polyadenylated. **A** Expression of MALNC in AML (*n* = 103) versus normal bone marrow (NBM, CD34^+^, (*n* = 11). Data shown as read counts log2(CPM + 1) from the KAW cohort (normalized by exon length) using box and whisker dot plots with interquartile range (IQR). **B** Genetic locus of *MALNC* (custom track, all lab-validated exons combined) in comparison to annotated *LOC105370601* gene with indicated exonic structure (RNA sequence XR_944092.2). Track data retrieved from NCBI (GRCh38.p13, Primary Assembly Homo sapiens, Chr14, and annotation release 109.20210514). **C** *(Track 1) MALNC* exonic structure as identified in this study spanning a genomic region of about 80 kb. *(Track 2)* Initially predicted genomic loci and exon structure of *XLOC_091701* on chromosome 14 by Cufflinks spanning 65,754 bp (chr14:14:83712431-83778185, GRChr37). *(Track 3-5)* Histograms and heatmap of summed coverage of read alignment in all ClinSeq AML samples (*n* = 325) within the Cufflinks predicted lncRNA locus +/- 1 kb. ClinSeq samples are sorted by mean coverage and brightest red equivalent to 323x coverage. Nucleotide coverage, represented by red bars, indicates exonic regions, while gap coverage, shown in blue, indicates intronic regions. *(Track 6-7)* Schematic overview of, to date, the three main identified isoforms of MALNC in HL60 and NB4 cell lines (corresponding to the three distinct transcription start sites (TSS) at exons 1.0, 1.1, and 1.2) and observed splicing pattern for the two alternative transcription termination sites (exon 7 and exon 10). Transcript sequences were amplified by primer walk and RACE, cloned and Sanger-sequenced. Sequences were then aligned against the reference genome using the hg19 assembly (Genome Reference Consortium Human Build 37, GRCh37). **D** Isoform usage as indicated by expression of MALNC by transcription start exons (exon 1.0, exon 1.1 and exon 1.2) among AML patients from KAW cohort (*n* = 72, only including samples with CPM > 0 in all exons). Data shown as read counts log2(CPM + 1) using box and whisker dot plots with interquartile range (IQR). **E** Isoform usage indicated by expression of MALNC by transcription termination exons (exon 7 and exon 10) among AML patients from KAW cohort (*n* = 58), including only samples with CPM > 0 in all exons. Data shown as read counts log2(CPM + 1), normalized by exon length using box and whisker dot plots with interquartile range (IQR). **F** Track view of CAGE-seq data in TSS of *MALNC* (chr14:83,711,285-83,737,170, GRCh37) for cell lines HL60, U937, monocytes, healthy CD34^+^ cells and K562. The locus is zoomed on *MALNC* start exon 1.1 (Ex1.1) and exon1.2 (Ex1.2). Data retrieved from FANTOM5 project. Data is scaled by group and was visualized using IGV. **G** ChIP-seq data track view of RNA polymerase 2 (Pol2A) binding and transcription factor binding sites of *c-Myc*, *Max, REST* and *PU.1* in transcription start locus of *MALNC* (chr14:83,711,285-83,737,170, GRCh37) for cell lines HL60 and K562. Data retrieved from ENCODE/HAIB (GSE32465) and visualized using IGV. P-values were determined by Student’s t-tests (1B;1E) and Two-way ANOVA followed by pairwise comparison testing (1D): ns- not significant, * < 0.05, ** < 0.01, *** < 0.001.

To identify novel lncRNAs potentially involved in AML biology, deep RNA sequencing was performed on a small discovery cohort comprising seven AML patient samples and 5 normal bone marrow (NBM) CD34^+^ mononuclear cell samples from healthy donors (Supplementary Table 1). De novo transcript identification and filtering were carried out as described in Supplementary Fig. 1A. Shortly, using in silico analysis [42], we selected RNAs longer than 200 bp with intergenic locations, a mean expression of ≥0.5 RPKM, and the presence of DNase I hypersensitivity sites and H3K4me3 histone marks (ENCODE data). Differential expression (DE) analysis between AML and NBM samples was performed using Cuffdiff (FDR < 0.05; fold change > |1.5 | ), identifying 136 DE transcripts, 83 upregulated and 53 downregulated in AML (Supplementary Fig. 1B; Supplementary Table 8). Further filtering was applied to retain transcripts with well-defined exon–intron boundaries, indicating proper splicing. Transcripts with abnormally long exons or single-exon structures, which may represent retained introns rather than truly spliced transcripts, were excluded. This yielded three candidate lncRNAs: XLOC_047983, XLOC_133217, and XLOC_091701. Among them, XLOC_091701 displayed the highest expression difference between AML and NBM samples (log_2_ fold change = 5.77, *P* < 0.001; Supplementary Table 8). Elevated expression of XLOC_091701 in AML cells was confirmed in a larger, internal validation cohort (KAW cohort: AML, *n* = 103; NBM CD34^+^, *n* = 11; *P* < 0.05; log_2_ fold change = 1.96; Supplementary Table 2; Fig. 1A). Therefore, XLOC_091701, located at chr14q.31.2, was selected for further functional characterization and named *myeloid and AML-associated intergenic long non-coding RNA* (MALNC), based on its characteristics described below.

To define the transcript structure of *MALNC*, the transcript prediction of Cufflinks (Supplementary Fig. 1C) was compared with read alignment from our population-based ClinSeq cohort of 325 AML patients (for characteristics, see Supplementary Table 2). Consistent overlap was found in terms of nucleotide coverage, supporting a transcript composed of 13 exons with a defined splicing pattern (Fig. 1B, C). Although a shorter transcript (*LOC105370601*) is annotated in NCBI, it only partially overlaps with *MALNC* (Fig. 1B; Supplementary Table 9). Next, we assessed MALNC expression across leukemic cell lines, observing the highest expression in the promyelocytic cell line HL60, followed by NB4, MOLM-13, U937, OCI-AML3 and OCI-AML2. In contrast, other AML cell lines and the CML cell line K562 showed no detectable MALNC expression (Supplementary Fig. 1D). Expression of MALNC in HL60 and U937 cells was further confirmed by the presence of Transcription Start Site (TSS) signals in Cap analysis of gene expression (CAGE) data sourced from FANTOM5 dataset, which captures the 5′ ends of transcripts to identify active promoters and TSSs at base-pair resolution (Supplementary Fig. 1E). Thus, in the following experiments, HL60, U937 and NB4 were used as MALNC-expressing cell models, while K562 and NBM CD34^+^ cells were used as negative controls.

To characterize the full-length transcript and splice-variants of MALNC, primer walking and rapid amplification of cDNA ends (RACE) were performed in HL60 and NB4 cells. This analysis identified three main isoforms, each starting from distinct first exons, designated as Ex1.0, Ex1.1 and Ex1.2 (Fig. 1C; Supplementary Fig. 2A). The first three exons are mutually exclusive and are followed by shared exons 2 and 3. Isoform Ex1.2 also includes an alternative exon 2.2, mutually exclusive with exon 2 and located within the intronic region between exons 2 and 3. Exon 2 also exhibited alternative splicing through partial intron retention, skipping a segment within the exon. Downstream of exon 3, several splice variants were observed, including isoforms that skip all exons between exons 3 and 10 (Fig. 1C). Transcripts may terminate at either exon 7 or exon 10, with variable inclusion of intermediate exons (Fig. 1C; Supplementary Fig. 2B, C). A comprehensive list of all exon sequences is provided in Supplementary Table 10.

RNA-seq data from the internal KAW-AML cohort showed predominant usage of transcription start exon 1.0 compared to exon 1.2, and more prominent usage of termination exon 7 over exon 10 in AML patients (Fig. 1D, E). Isoform usage was further investigated in APL patients using RNA-seq data from the KAW cohort, as well as in healthy promyelocytes from publicly available datasets [43]. In APL patients (*n* = 5), transcription was predominantly initiated from exon 1.1 compared to exons 1.0 and 1.2, while no significant difference was observed in termination between exons 7 and 10 (Supplementary Fig. 2D). Similarly, in healthy promyelocytes (*n* = 8), exon 1.1 was the only transcription start exon expressed, and no differential usage was detected between termination exons 7 and 10 (Supplementary Fig. 2E). 3´ RACE analysis showed then presence of a natural poly(A) tail at both transcription termination sites (Supplementary Fig. 2B, C). Furthermore, CAGE-seq data showed that *MALNC* is longer (Fig. 1F; Supplementary Fig. 2F) and more complex than the currently annotated *LOC105370601*.

Finally, using publicly available DNase-seq, ATAC-seq and ChIP-seq data from ENCODE, we identified DNase I hypersensitivity sites and chromatin accessibility at the TSS of *MALNC* in HL60, NB4, and monocytes, but not in K562 or CD34^+^ cells (Supplementary Fig. 2G). RNA Polymerase II occupancy was detected at the TSSs in HL60, but not in K562 (Fig. 1G). Similarly, H3K4me3 marks were present in HL60 and NB4, but not in K562, and the active enhancer mark H3K27ac was present in HL60 only (Supplementary Fig. 2H). Together, these findings indicate active transcription of the *MALNC* locus in HL60 and NB4 AML cell lines, but not in K562 or normal CD34^+^ cells. ChIP-seq data further revealed c-MYC and MAX binding to the *MALNC* locus in NB4 cells, and PU.1 binding in HL60, but not K562 cells (Fig. 1G). This data suggests that c-MYC, MAX, and PU.1 may be involved in the upstream regulation of MALNC. Together, these findings establish MALNC as a polyadenylated lncRNA that is specifically upregulated and actively transcribed in AML, particularly in promyelocytic subtypes, and potentially regulated by key myeloid transcription factors.

### MALNC is a non-coding RNA, specifically expressed in bone marrow and localized both in the nucleus and cytoplasm

**Fig. 2:**
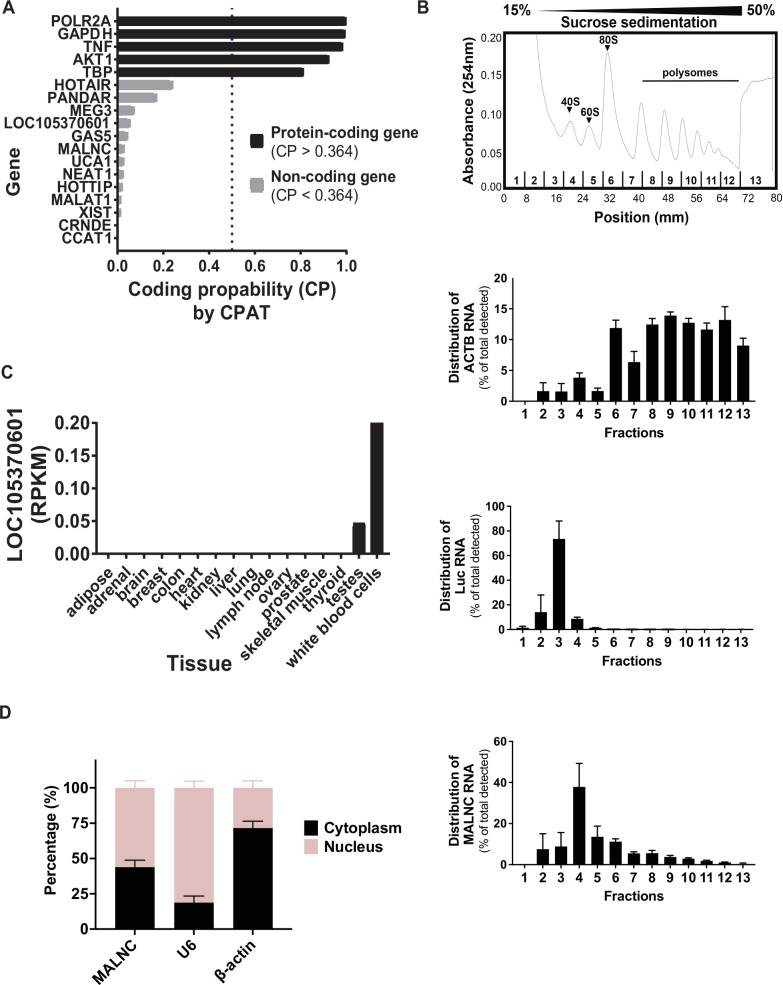
MALNC is a non-coding RNA specifically expressed in bone marrow. **A** In silico coding probability prediction of currently annotated lncRNA LOC105370601 and the longer MALNC RNA transcript in comparison to well-investigated coding (TNF, POLR2A, GAPDH, AKT1, TBP) and non-coding (HOTAIR, PANDAR, MEG3, XIST, NEAT1, HOTTIP, MALAT1, CRNDE, GAS5, UCA1, CCAT1) RNA transcripts. Coding probability was determined by CPAT web tool application (Coding Potential Assessment tool, coding cut-off >0.364). **B** Non-coding potential of MALNC was determined by its association with ribosomes. Cell lysates from HL60 cells were analyzed by 15–50% sucrose density gradient fractionation. RNA was extracted from each gradient fraction and expression of ACTB RNA, spike Luciferase RNA (Luc) and MALNC RNA was determined by RT-qPCR. *(Top)* Sucrose gradient absorbance profile (254 nm) from HL60 cells indicating the localization of free RNA, ribosomal subunits (40S and 60S), monosomes (80S) and polysomes along the sampled sucrose gradient fractions (1–13). One representative absorbance profile is shown. *(Bottom)* Bar charts presenting the distribution of co-sedimented *ACTB*, Luc and MALNC RNA transcripts in HL60 from each sucrose gradient fraction as percent from total RNA detected by RT-qPCR. Data is shown as mean with SEM of 3–4 fractionations and was normalized to spiked-in XENO RNA. **C** RNA expression of lncRNA LOC105370601 (RPKM, Total RNA-seq) in 16 different normal tissue types (Illumina bodyMap2 transcriptome data, BioProject: RJEB2445). **D** Level of RNA from nuclear and cytoplasmic fractions in HL60 cells. Data are shown from three biological experiments. MALNC localization was compared to ACTB RNA, which is predominantly exported to the cytoplasm, and U6 RNA, which is predominantly located in the nuclear compartment.

In silico predictions classified MALNC as non-coding (Fig. 2A; Supplementary Fig. 3A–C). To confirm this in vitro, we performed polysome profiling in both HL60 cells and NB4 cells. Whereas the protein-coding ACTB mRNA was predominantly associated with polysomes, indicative of active translation, MALNC RNA was predominantly localized in ribosome-free RNA fractions, supporting its classification as a non-coding transcript (Fig. 2B; Supplementary Fig. 3D). Furthermore, secondary structure prediction of MALNC revealed a fold-rich RNA architecture (Supplementary Fig. 3E), suggesting potential for diverse biomolecule binding.

MALNC was found to be primarily expressed in bone marrow (Fig. 2C; Supplementary Fig. 4A, B) while analysis of evolutionarily conserved regions (ECR) [44] indicated that *MALNC loci* may be conserved among primates, with the most conserved loci localizing in regulatory regions (Supplementary Fig. 4C). To explore MALNC subcellular localization, we performed cell fractionation in HL60 cells and found that MALNC is present in both the nucleus and cytoplasm (Fig. 2D). These findings further support MALNC as a structurally complex, non-coding RNA with potential regulatory roles in both nuclear and cytoplasmic compartments.

### MALNC associates with APL and IDH2^R140^/NPM1-mutated AML

**Fig. 3:**
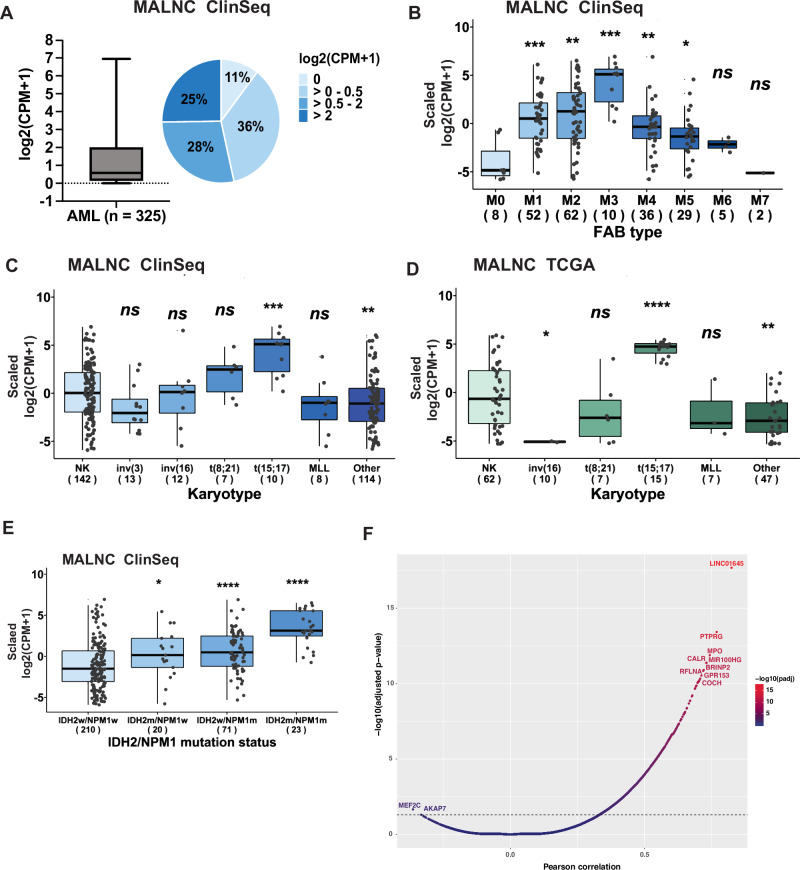
MALNC associates with APL and *IDH2*^*R140*^*/NPM1*-mutated AML. **A** Box plot, presenting the distribution, and pie chart, presenting the percentage, of MALNC counts among AML patients in the ClinSeq cohort (*n* = 325) classified as MALNC non-expressors (log2(CPM + 1) = 0 and log2(CPM + 1) > 0–0.5) and MALNC expressors (log2(CPM + 1) > 0.5 – 2 and log2(CPM + 1) > 2). **B** Expression of MALNC among AML FAB groups in the ClinSeq cohort (*n* = 204). Normalized counts (log2(CPM + 1)) were scaled and shown as box and whisker dot plot with interquartile range (IQR). The sample number is indicated in parentheses (n). P-value determined by Kruskal–Wallis test followed by Dunn´s pairwise comparison test. **C**, **D** Expression of MALNC among normal karyotype AML and AML with chromosomal aberrations (inv(3), inv(16), t(8;21), t(15;17), MLL, Other) in the ClinSeq cohort (n = 306) and TCGA cohort (*n* = 148). Normalized counts (log2(CPM + 1)) were scaled and shown as box and whisker dot plot with interquartile range (IQR). The sample number is indicated in parentheses (n). P-value determined by Kruskal–Wallis test followed by Dunn´s pairwise comparison test. **E** Distribution of MALNC expression in patients with *NPM1/IDH2*^*R140*^ wild-type AML (IDH2w/NPM1w), *NPM1* wild-type and mutated *IDH2*^*R140*^ (IDH2m/NPM1w), mutated *NPM1*, wild-type *IDH2*^*R140*^ (IDH2w/*NPM1*m) AML and co-occurring *NPM1/IDH2*^*R140*^ mutation (IDH2m/NPM1m) in the ClinSeq cohort (n = 324). Normalized counts (log2(CPM + 1)) were scaled and shown as box and whisker dot plot with interquartile range (IQR). The sample number is indicated in parentheses (n). P-value determined by Kruskal–Wallis test followed by Dunn´s pairwise comparison test. **F** Pearson correlation analysis between MALNC expression and gene expression in the ClinSeq cohort (*n* = 325). The top ten positively correlated genes are shown in red, and the 2 negatively correlated genes are shown in blue. FAB French-American-British-AML classification, NPM1 Nucleophosmin 1, IDH2 Isocitrate dehydrogenase, wt wild-type, mut mutated, APL acute promyelocytic leukemia.

We next examined MALNC expression across AML patient populations. MALNC expression showed consistent patterns in two independent AML cohorts, our own ClinSeq cohort (*n* = 325) and the publicly available TCGA-LAML cohort (*n* = 151) (Supplementary Table 2), with expression detected in approximately one-third to half of the patients (Fig. 3A; Supplementary Fig. 5A). Notably, MALNC expression displayed a striking correlation with the presence of the APL-specific *PML-RARA* fusion gene, with all APL patients exhibiting high MALNC levels (Fig. 3B–D; Supplementary Fig. 5B–D). Furthermore, MALNC expression correlated positively with mutant *NPM1* (*P* < *0.0001*), *IDH2*^*R140*^ (*P* < *0.0001*), *FLT3*-ITD (*P* < *0.001*) and *STAG1* (*P* < *0.05*), and negatively with *RUNX1* mutations (*P* < *0.0001*) (Supplementary Fig. 5E). Similarly to APL patients, patients with co-occurring *NPM1* and *IDH2*^*R140*^ mutations, but not other *IDH* variants, also showed markedly elevated MALNC expression (Fig. 3E; Supplementary Fig. 5C, D; 6A–C).

To identify genes co-expressed with or inversely correlated to MALNC, we performed a correlation analysis between MALNC expression and the expression levels of all other genes profiled by RNA-seq in the ClinSeq cohort (*n* = *325)*. The results are summarized in Fig. 3F, which highlights the top ten positively correlated genes and the two most negatively correlated genes. A comprehensive ranking of all genes is provided in Supplementary Table 11. Notably, among the top co-expressed genes were *myeloperoxidase (MPO)* and *calreticulin (CALR)*, both known to be highly expressed during the promyelocyte stage of myeloid differentiation. Consistently, during normal hematopoiesis, MALNC was minimally expressed in normal CD34+ progenitor cells (Fig. 1A, F; Supplementary Fig. 2F, G), peaked at the promyelocytic stage, and gradually decreased with granulocytic maturation (Supplementary Fig. 6D–F). Together, these data suggest that MALNC expression is associated with specific AML subtypes and stages of myeloid differentiation.

### MALNC decreases at ATRA-induced myeloid differentiation

**Fig. 4:**
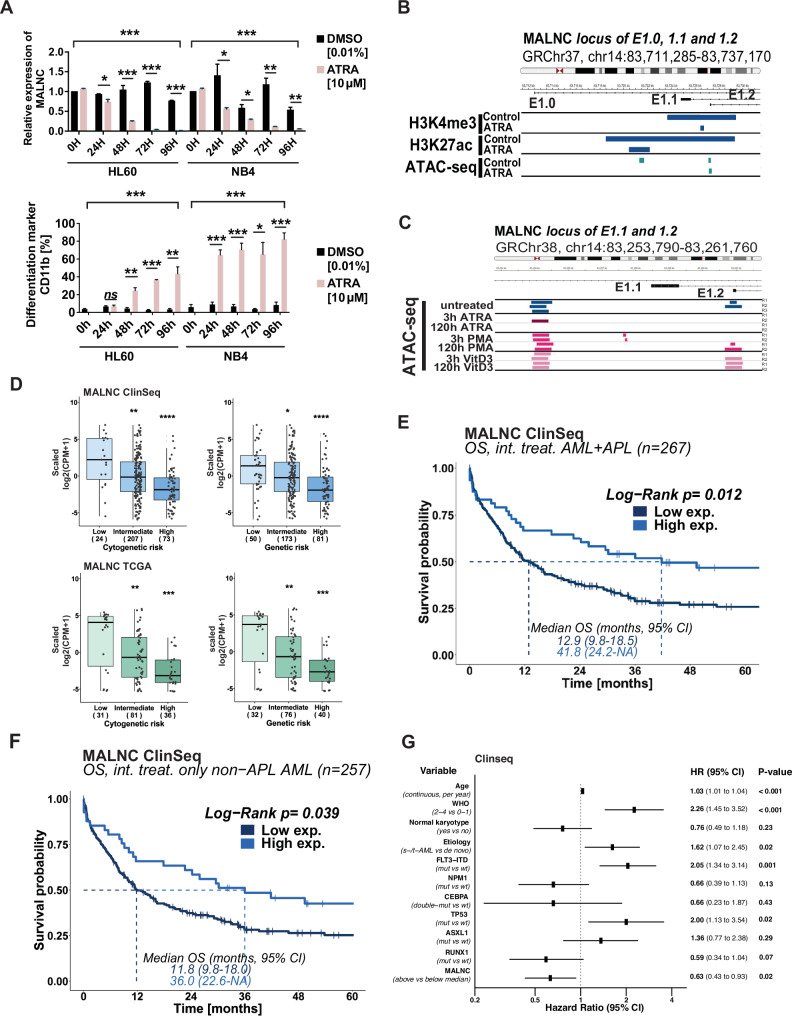
MALNC decreases during ATRA-induced myeloid differentiation and associates with better OS in AML patients. **A***(Top)* Relative expression of MALNC determined by RT-qPCR in HL60 and NB4 cells during all-*trans* retinoic acid-induced differentiation (10 µM ATRA, 96 h). Data are shown as means ± SEM from three biological experiments, and relative to time point 0 h and normalized towards endogenous control TBP. *(Bottom)* Level of differentiation marker CD11b during ATRA and vehicle (DMSO 0.01%) treated HL60 and NB4 cells. The percentage of CD11 b-positive cells relative to time point 0 h was determined by flow cytometry up to 96 h. Data are shown as means ± SEM from three biological experiments. P-values were determined by Students t-tests: ns- not significant, *p-value < 0.05, **p-value < 0.01, ***p-value < 0.001. **B** Peaks of histone modifications H3K4me3 and H3K27ac and chromatin accessibility (ATAC-seq) around the TSS of *MALNC* gene (chr14:83,711,285-83,737,170, GRCh37) for ATRA (1 µM, 96 h) and vehicle control (ethanol) treated HL60. Data retrieved from ENCODE (GSE93994 and GSE93993). **C** Chromatin accessibility (ATAC-seq) in the TSS of *MALNC* (chr14:83, 253,790-83,261,760 GRCh38) for untreated HL60 as well as ATRA, phorbol 12-myristate-13-acetate (PMA) and Vitamin D3 (VitD3) treated HL60. Data retrieved from ENCODE (GSE79019) **D** Expression of MALNC among cytogenetic and genetic risk groups in the *(top)* ClinSeq cohort (*n* = 304) and *(bottom)* TCGA-LAML cohort (*n* = 148). Normalized counts (log2(CPM + 1)) were scaled and shown as box and whisker dot plots with interquartile range (IQR). The sample number is indicated in parentheses (n). P-value determined by Kruskal–Wallis test and then followed by Dunn´s pairwise comparison test. **E**, **F** Kaplan-Meier survival curves showing overall survival (OS) of AML patients stratified by MALNC expression (CPM cut-off by median). Data is shown in the ClinSeq-AML cohort of intensively treated patients, including APL (*n* = 267) or excluding APL patients (*n* = 257). P-value determined by log-rank test. Data was censored at the time of allogeneic stem cell transplantation (allo-HSCT), if applicable. **G** Multivariate analysis was performed on non-APL AML patients from ClinSeq cohort (*n* = 257). FAB French-American-British-AMLclassification, NPM1 Nucleophosmin 1, IDH2 Isocitrate dehydrogenase, wt wild-type, mut mutated, APL acute promyelocytic leukemia.

Next, we examined MALNC expression and chromatin landscape during myeloid differentiation of HL-60 and NB4 cells. Consistent with MALNC downregulation during normal myelopoiesis, ATRA-induced granulocytic differentiation caused a gradual decrease in MALNC expression, with undetectable expression levels at the most differentiated stage, as assessed by FACS and RT-qPCR analyzes (Fig. 4A; Supplementary Fig. 7A). This was supported by publicly available (ENCODE) ChIP-seq and ATAC-seq data from HL60 cells, which showed reduced levels of the active enhancer marks H3K4me3 and H3K27ac, along with decreased chromatin accessibility at the *MALNC* locus during ATRA-exposure (Fig. 4B, C). In contrast, MALNC expression did not change during Vitamin-D3-induced monocytic differentiation (Supplementary Fig. 7B), nor did Vitamin-D3 monocytic differentiation or megakaryocytic differentiation by PMA alter chromatin accessibility (Fig. 4C). These findings indicate that *MALNC* undergoes transcriptional repression specifically during granulocytic differentiation.

### MALNC expression correlates to improved AML survival independently of APL and other prognostic factors

To gain insight into the clinical relevance of MALNC, we further examined whether its expression in AML is associated with disease biology and patient outcomes. MALNC expression correlated with low-risk cytogenetic groups in both the Clinseq and TCGA cohorts (*P* < *0.05*; Fig. 4D). No significant correlation was observed with AML etiology, karyotype, blood parameters, or other recurrent AML mutations (Supplementary Fig. 7C, D, 8A). Notably, high MALNC expression correlated significantly with better overall survival (OS) in both AML cohorts (ClinSeq-AML: *P* = *0.012*; TCGA-cohort: *P* = *0.014*; Fig. 4E, Supplementary Fig. 8B). In addition, our internal ClinSeq cohort, which comprises a broader and larger set of AML patients compared to the TCGA cohort, also showed improved OS when APL patients were excluded (*P* = *0.039*; Fig. 4F). Additionally, in a multivariable analysis, MALNC expression was significantly associated with better overall survival, also independently of other prognostic factors (HR = 0.63, 95% CI: 0.43–0.93; *P* < 0.02; Fig. 4G). These findings suggest that MALNC may serve as a potential prognostic biomarker in AML.

### MALNC knockout changes the expression of genes related to retinoic acid signaling and impairs cell viability and colony formation

**Fig. 5:**
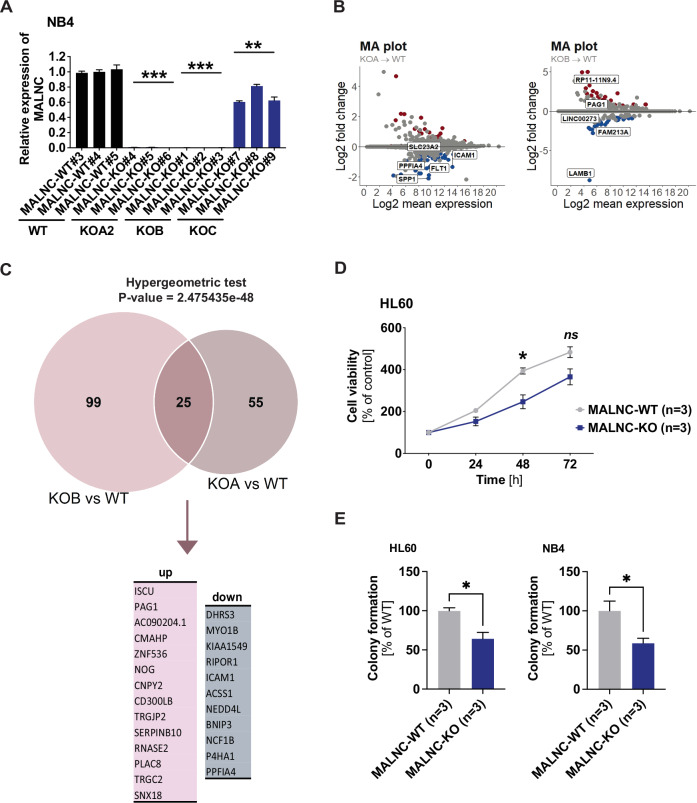
*MALNC* knockout alters the expression of genes associated with differentiation and retinoic acid signaling and impairs cell viability. **A** Relative expression (determined by RT-qPCR) of MALNC in NB4 WT and KO clones (three clones each) used for RNA-sequencing. Data are shown as means ± SEM from three biological experiments relative to *MALNC*^WT#3^ clone expression and normalized towards endogenous control TBP. **B** MA-plot showing the shrunken log2 fold changes (LFC) of differentially expressed genes (DEGs) between *(top) MALNC*^KOA^ and *MALNC*^WT^ and *(bottom) MALNC*^KOB^ and *MALNC*^WT^. **C** Venn diagram showing the overlap of DEGs between *MALNC*^KOA^ vs *MALNC*^WT^ and *MALNC*^KOB^ vs *MALNC*^WT^ (*n* = 25). Lists indicate which of those identified differentially expressed genes are shared up-regulated (*n* = 14) and shared down-regulated (*n* = 11) in *MALNC*^KOA^ vs *MALNC*^WT^ and *MALNC*^KOB^ vs *MALNC*^WT^. A hypergeometric test was performed to assess the overlap between DEGs in MALNC KOA and KOB. **D** Cell viability in HL60-*MALNC*^KO^ (*n* = 3), *MALNC*^WT^ (*n* = 3) cells. Cell viability in percent of control (4 h) was measured by WST-8 reagent up to 72 h. Data is shown as means ± SEM from three biological experiments. **E** Relative colony formation between *MALNC*^KO^ and *MALNC*^WT^ cells in both HL60 and NB4 cells. Data is shown as means ± SEM from three biological experiments using three KO clones and three WT clones, respectively. The percentage of colony formation is relative to WT. P-values were determined by Student’s t-test: ns, not significant, * < 0.05, ** < 0.01, *** < 0.001.

To further study MALNC´s role, CRISPR-Cas9 editing was used to generate several *MALNC*-knockout (KO) clones in NB4 and HL60 AML cell lines by deleting various TSSs and the full transcript locus (Supplementary Fig. 9A–C). In KOA, Ex1.1 and Ex1.2 were deleted, in KOB, Ex1.0 was deleted in addition to Ex1.1 and Ex1.2, while in KOC, the entire transcript locus was deleted. KOA and KOB single-cell clones with complete loss of MALNC expression were selected for further analysis, whereas KOC clones showed only a 50% reduction in expression due to the lack of homozygous deletions (Fig. 5A, Supplementary Fig. 9D). This reduced editing efficiency, observed in KOC clones, can be attributed to the large size of the targeted region ( > 70 kilobases) and the associated complexity of DNA repair mechanisms.

RNA-seq of NB4-*MALNC*^KOA^ clones versus NB4-*MALNC*^WT^ identified 22 genes that were upregulated and 58 downregulated, while RNA-seq of NB4-*MALNC*^KOB^ versus NB4-*MALNC*^WT^ exhibited 55 upregulated and 69 downregulated genes (Fig. 5B; Supplementary Fig. 9E). In total, 80 and 124 genes were differentially expressed in NB4-*MALNC*^KOA^ and NB4-*MALNC*^KOB^ cells, respectively (Supplementary Tables 12, 13). Of these, 25 genes were commonly deregulated in both KO types and with the same direction of regulation: 14 upregulated and 11 downregulated (Fig. 5C, Supplementary Table 14). This overlap was statistically significant (*P* = 2.47 × 10^−48^, hypergeometric test), despite KOA and KOB targeting only partially overlapping TSSs. Among the shared differentially expressed genes (DEGs), *DHRS3* and *ZNF536*, both components of the retinoic acid pathway, were further validated by RT-qPCR in both KO types (Supplementary Fig. 9F), suggesting that MALNC modulates retinoic acid signaling.

To characterize the functional consequences of MALNC loss, we next assessed proliferation and colony-forming capacity. *MALNC* knockout significantly decreased proliferation, as measured by viability assays of HL60-*MALNC*^KO^ cells compared to HL60-*MALNC*^WT^ (*P* < *0.05*, at 48 h) (Fig. 5D), while a similar trend, although not statistically significant, was found in NB4-*MALNC*^KO^ (Supplementary Fig. 10A). Furthermore, granulocyte-macrophage colony formation was also significantly decreased in *MALNC*^KO^ clones from both HL60 and NB4 cells (Fig. 5E). While colony-forming assays showed clear differences between *MALNC*^KO^ and *MALNC*^WT^ cells, cell cycle and apoptosis analyzes showed no significant difference in unexposed cells (Supplementary Fig. 10B, C). These findings demonstrate that MALNC regulates a distinct set of genes, including components of the retinoic acid signaling pathway, and is required for sustaining AML cell proliferation and colony-forming capacity.

### Loss of MALNC amplifies ATRA-mediated myeloid differentiation and increases the expression of ATRA-induced genes

**Fig. 6:**
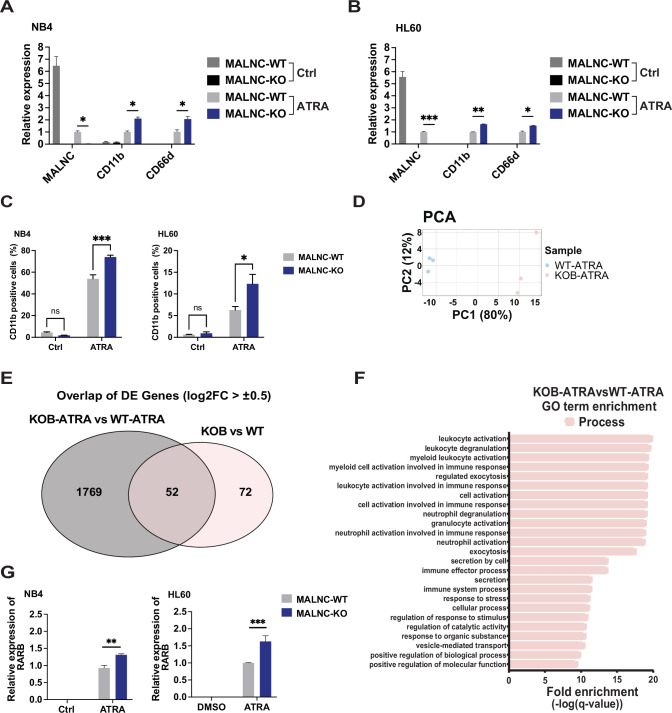
MALNC affects ATRA-induced myeloid differentiation. **A**, **B** Relative expression (RT-qPCR) of *MALNC, CD11b and CD66d* in NB4- and HL60- *MALNC*^WT^ and *MALNC*^KO^ clones after ATRA-induced differentiation (1 µM ATRA, 72 h). Data are shown as means ± SEM from three biological experiments relative to *MALNC* expression in ATRA-treated WT cells and normalized towards endogenous control TBP. **C** Percentage of CD11b positive cells in NB4- and HL60- *MALNC*^WT^ and *MALNC*^KO^ cells. Cells were treated with either vehicle control (DMSO, 0.01%) or ATRA (1 µM) for 72 h, and data were obtained by flow cytometry. Data are presented as means ± SEM from three biological replicates. **D** Principal component analysis (PCA) of RNA-sequencing samples *MALNC*^KOB-ATRA^ and *MALNC*^WT-ATRA^. **E** Venn diagram showing the overlap between genes DE in NB4 *MALNC* KOB cells versus NB4 *MALNC* WT cells under ATRA treatment (RNA-seq: *n* = 1,821) and genes DE in NB4 *MALNC* KOB cells vs WT cells at basal level (RNA-seq: *n* = 124). **F** Gene ontology (GO) biological process term enrichment analysis on differentially expressed genes dysregulated among KOB^ATRA^ (ATRA-treated NB4 *MALNC*-KO#2, KOB) and WT^ATRA^ (ATRA-treated NB4 *MALNC*^WT#4^). GO and pathway analysis were performed using GOrilla (Gene Ontology enrichment analysis and visualization tool). Analysis was performed using target gene list against background gene list with a q-value cut-off of 0.01. The target list consisted of genes (*n* = 1,821) differentially expressed between KOB^ATRA^ (ATRA-treated NB4-*MALNC*^KO#2^) versus WT^ATRA^ (ATRA-treated NB4 *MALNC*^WT#4^), KOB^ATRA^ vs WT^ATRA^. **G** Relative expression (RT-qPCR) of RARB in NB4- and HL60- *MALNC*^WT^ and -*MALNC*^KO^ clones after ATRA-induced differentiation (1 µM ATRA, 72 h). Data are shown as means ± SEM from three biological experiments relative to RARB expression in WT control cells and normalized towards endogenous control TBP. **A**, **B**; **C**, **G** P-values were determined using Two way ANOVA followed by pairwise comparison testing: ns – not significant, ns, not significant, * < 0.05, ** < 0.01, *** < 0.001.

Given the involvement of MALNC in retinoic acid signaling and its impact on AML cell viability, we next investigated the functional effects of MALNC loss on granulocytic differentiation. *MALNC*^KO^ significantly sensitized both NB4 and HL60 cells to ATRA-induced differentiation compared to their *MALNC*^WT^ counterparts, as demonstrated by increased expression of granulocytic differentiation markers. RT-qPCR analysis revealed a significant increase in the mRNA levels of granulocytic markers CD11b and CD66d in NB4 and HL60 *MALNC* KO cells compared to WT cells following ATRA treatment (Fig. 6A, B). In addition, surface expression of CD11B protein was also significantly higher in both NB4-*MALNC*^KO^ and HL60-*MALNC*^KO^ cells during ATRA exposure, as shown by FACS analysis (Fig. 6C). Furthermore, RNA sequencing was performed on ATRA-treated NB4-*MALNC*^WT^ (*MALNC*^WT-ATRA^) versus ATRA-treated NB4-*MALNC*^KO^ cells carrying all three TSS deletions (*MALNC*^KOB-ATRA^) (Fig. 6D), identifying 1821 DEGs (adjusted *P* < 0.05, Log2FC > |0.5 | ) (Supplementary Fig. 10D; Supplementary Table 15). By overlapping the 1,821 differentially expressed genes identified after ATRA treatment (*MALNC*^KOB-ATRA^ vs *MALNCWT*^-ATRA^) with those differentially expressed at basal levels (*MALNC*^KOB^ vs *MALNC*^WT^), 52 genes were found to be commonly deregulated under both conditions (Fig. 6E, Supplementary Table 16). Further analysis of DEGs overlaps across multiple conditions, including genes regulated by ATRA in the presence and absence of MALNC, as well as genes regulated both by ATRA alone and by MALNC with or without treatment, are presented in Supplementary Fig. 10E–F and detailed in Supplementary Tables 17–20.

Gene ontology (GO) term analysis of DEGs between NB4-*MALNC*^WT-ATRA^ and NB4-*MALNC*^KOB-ATRA^ primarily identified terms related to myeloid differentiation (Fig. 6F; Supplementary Figs. 11A, 10B; Supplementary Tables 21–23). Among the DE genes, the key retinoic acid response gene *RARB* [45] was found to be more expressed in NB4-*MALNC*^KO^ cells upon ATRA treatment (Supplementary Fig. 11C). This upregulation was further confirmed by RT-qPCR in both NB4 and HL60 ATRA-treated knockout cells (Fig. 6G). These results indicate that MALNC loss enhances the transcriptional and phenotypic response to ATRA, promoting granulocytic differentiation through upregulation of retinoic acid-responsive genes, including *RARB*.

### MALNC binds to the chromatin of genes related to the Rho GTPase and retinoic acid pathways and modulates drug sensitivity

**Fig. 7:**
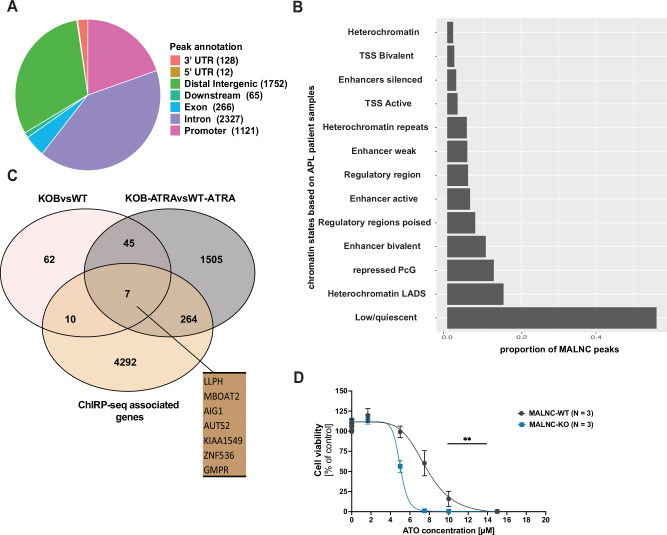
*MALNC* physically binds to the chromatin and depletion of MALNC sensitizes AML cells to arsenic trioxide treatment. **A** Venn diagram showing the percentage of MALNC binding sites among different chromatin regions. 3’ UTR (Untranslated Region): Peaks overlapping the 3’ UTR of transcripts. 5’ UTR: Peaks overlapping the 5’ UTR of transcripts. Distal Intergenic: Peaks located in intergenic regions, farther than 3 kb from any annotated transcription start site (TSS) or transcription end site (TES). Downstream: Peaks located within 3 kb downstream of the TES. Exon: Peaks overlapping coding exons, excluding UTRs. Intron: Peaks located within intronic regions. Promoter: Peaks located within 3 kb upstream to 3 kb downstream of the TSS. **B** Enrichment of MALNC ChIRP-seq peaks across different chromatin states, based on H2AZ, H3K4me1, H3K4me3, H3K9me2, H3K9me3, H3K18ac, H3K27me3, H3K27ac ChIP-seq data from 5 APL patients. **C** Venn diagram showing the overlap between genes associated to MALNC chromatin binding sites (ChIRP-seq: *n* = 4573), genes differentially expressed in NB4 *MALNC* KOB cells (RNA-seq: *n* = 124) and genes differentially expressed in NB4 *MALNC* KOB cells treated with ATRA (RNA-seq: *n* = 1821). **D** Dose-response curve of NB4-*MALNC*^WT^ clones (*n* = 3) and NB4-*MALNC*^KOB^ clones (*n* = 3) with a serial dilution of arsenic trioxide starting at 15 µM and luminescence measurement of viable cells at 72 h post-treatment using CellTiterGlo assay. Data are shown from three biological experiments. Student *t* test was used for statistical analyzes. Statistical significance is indicated as follows: P-values were determined by Student’s *t* test: ** < 0.01.

Chromatin isolation by RNA precipitation sequencing (ChIRP-seq) was performed in NB4 cells in order to identify MALNC chromatin interaction sites. DNA sequencing identified 5,671 genomic loci bound by MALNC (Supplementary Table 24), with the majority located in introns (2327 peaks), followed by distal intergenic regions (1752 peaks) and promoters (1,121 peaks) (Fig. 7A). In primary APL patient cells (KAW cohort; *n* = 5), MALNC was shown to preferentially bind to repressive chromatin states related to polycomb targets compared to active chromatin (Fig. 7B). *GO* and Reactome pathway analysis of MALNC binding peaks showed an enrichment of terms related to the Rho GTPase pathway (Supplementary Fig. 11D, E). Additionally, MALNC was found to bind several genes involved in the retinoic acid pathway, including *RARA* at the promoter region, and *STAT1* and *RXRA* genes within their intronic regions (Supplementary Table 24).

To identify potential direct targets of MALNC, we first overlapped genes differentially expressed in NB4-*MALNC* knockout (KOB) cells under basal conditions (RNA-seq; *n* = 124) with those bound by MALNC, as identified by ChIRP-seq (genes with at least one MALNC-associated peak; *n* = 4573; Supplementary Table 25). This analysis revealed 17 overlapping genes (Fig. 7C). Next, to identify MALNC chromatin interactions at a basal state that might modulate gene expression changes that become effective during ATRA cell differentiation, we overlapped the ATRA-responsive genes in NB4-*MALNC* KOB cells (RNA-seq; *n* = 1821) with the MALNC-bound genes. This revealed 271 overlapping genes (Fig. 7C). Among these, seven genes were common across all three datasets: (i) MALNC-bound genes, (ii) DEGs in KOB vs. WT under basal conditions, and (iii) DEGs in KOB vs. WT after ATRA treatment (Fig. 7C). Notably, this overlapping set includes *ZNF536*, a gene functionally linked to the retinoic acid pathway (Supplementary Table 24).

Finally, to assess whether MALNC influences drug sensitivity, we performed a high-throughput drug sensitivity and resistance testing (DSRT) screen on three NB4-*MALNC*^KO^ clones and three NB4-*MALNC*^WT^ clones using a comprehensive panel of 525 cancer-related drugs. Drugs were filtered based on the Drug Sensitivity Score (sDSS), which reflects the difference in drug response between *MALNC* KO and *MALN*C WT cells, using a threshold of ±1 (Supplementary Table 27). Interestingly, arsenic trioxide (ATO), a standard therapy for APL when combined with ATRA, showed a trend toward increased sensitivity in NB4-*MALNC*^KO^ cells in the DSRT screen (sDSS = 1.633). This observation was further confirmed in vitro, where NB4 cells lacking *MALNC* expression exhibited significantly higher sensitivity to arsenic trioxide compared to *MALNC*-expressing cells (Fig. 7D). In sum, these findings suggest that MALNC exerts its regulatory effects at least in part through direct chromatin interactions and that its loss enhances sensitivity to arsenic trioxide (ATO), a key therapeutic agent in APL.

## Discussion

In this study, we report the discovery and characterization of MALNC, a novel lncRNA overexpressed in AML. Our findings reveal MALNC as a multi-exonic lncRNA with elevated expression in specific AML subclasses, particularly in APL and non-APL AML with *NPM1/IDH2*^*R140*^co-mutations, which account for 10% and 5% of AML patients, respectively [25, 46]. This despite the fact that APL and *NPM1/IDH2*^*R140*^-co-mutated AML constitute two different AML subtypes, both genetically and morphologically. This pattern of subtype-specific expression is in line with previous reports of lncRNAs such as HOXB-AS3 and LONA which are associated with mutant *NPM1* AML [29, 30]. Thus, certain lncRNAs appear to be associated with specific genetic subgroups of AML, although their functional roles within these subtypes remain to be elucidated.

Interestingly, MALNC is dynamically expressed in normal myelopoiesis, with minimal levels in immature CD34^+^ cells, peaking at the promyelocytic stage, and declining during granulocytic maturation, a pattern also observed during differentiation of promyelocytic leukemia cell lines. Clinically, higher *MALNC* expression significantly correlated with improved overall survival in AML. While this association may partly reflect correlations with established favorable-risk markers such as the *PML-RARA* fusion and mutant *NPM1* [21, 47], MALNC expression remained significantly associated with better prognosis even after adjusting for these and other known favorable-risk genetic factors. This suggests that MALNC may serve as a broader prognostic factor, potentially identifying additional subsets of AML patients with favorable outcomes beyond those defined by current genetic markers. Somewhat counterintuitively, *MALNC* KO impaired AML cell proliferation and colony formation, while enhancing ATRA-induced granulocyte differentiation. This implies that MALNC may play a role in maintaining cells in a less differentiated and more proliferative state, and aligns with the observation that certain proliferative genetic events in AML also confer a favorable prognosis [48–50]. In addition, a more proliferative state is often associated with a better response to chemotherapy.

Knocking-out *MALNC* significantly impaired colony formation and enhanced ATRA-induced differentiation, which further supports the notion that MALNC keeps promyelocytic leukemic cells in a more proliferative and undifferentiated state. Drug screening data revealed that MALNC influences the sensitivity of AML cells to arsenic trioxide, with a significant difference observed between *MALNC* WT and KO cells. This highlights that lncRNAs can influence sensitivity to specific antileukemic substances, thereby underscoring their potential utility as biomarkers for drug sensitivity and/or their usage as targets for novel treatment strategies in the future. Furthermore, understanding the functions of lncRNAs could help elucidate the mechanisms of action of specific drugs and explain the heterogeneity in drug responses among patients. In the case of MALNC, its impact on ATO should be further evaluated in combination with other anti-leukemic agents such as ATRA, which together constitute the standard treatment for APL. This would help determine whether MALNC expression levels might influence treatment response in APL patients.

Whole transcriptome sequencing of *MALNC* knock-out cells at baseline and during ATRA-induced differentiation revealed DEGs involved in the RA pathway and leukocyte differentiation. Notably, the RA-related genes *DHR3* and *ZNF536* were differentially expressed at baseline, and the key ATRA-induced gene *RARB* was more strongly induced in *MALNC*^KO^ cells upon treatment. ChIRP-seq analysis further showed that MALNC binds directly to several RA pathway genes (*RARA*, *STAT1*, *RXRA*, *ZNF536*) and to genes involved in the Rho GTPase pathway, which is implicated in AML and APL biology [47–49]. These findings suggest that MALNC may modulate transcriptional responses to ATRA by directly interacting with chromatin at key regulatory loci.

The transcriptional effects of MALNC were more pronounced during ATRA treatment than at baseline, which indicates that MALNC especially exerts its effects during granulocytic maturation. In the nucleus, lncRNAs like MALNC can regulate transcription via diverse mechanisms. They may recruit chromatin-modifying complexes that alter local chromatin structure, either activating or repressing gene expression. They can also form RNA-DNA triplex structures that impact transcription factor (TF) binding at specific loci, mediate TF sequestration, or serve as scaffolds to bring chromatin-modifying proteins to target sites. Notably, subcellular fractionation analyzes revealed that MALNC is not exclusively nuclear but also localizes to the cytoplasm. This observation raises the possibility that MALNC may exert additional functions beyond nuclear transcriptional regulation, potentially affecting mRNA stability, translation, or interacting with RNA-binding proteins and microRNAs. These regulatory functions are often highly context-dependent, influenced by factors such as cell type, lncRNA isoforms, subcellular localization and binding properties [6, 29]. Therefore, additional investigations into chromatin binding and alternative modes of action are needed to fully understand how MALNC exerts its biological effects.

As for the upstream regulation of *MALNC*, we hypothesize that MALNC, like other lncRNAs [51], may be transcriptionally controlled by c-Myc. Supporting this, c-Myc was observed at the *MALNC* locus in cells expressing the lncRNA, but not in *MALNC-*negative cells. Given c-Myc’s well-known role as an oncoprotein and regulator of gene transcription [52–54], this regulatory link warrants further investigation.

Overall, in this study, we have identified and characterized MALNC as a novel lncRNA specifically expressed in APL and *NPM1/IDH2*^*R140*^ co-mutated AML. MALNC influences key leukemic processes including cell proliferation, cell differentiation and drug responses. While our findings point to several upstream and downstream regulators of MALNC, further studies are needed to fully elucidate its molecular mechanisms and evaluate its potential as a prognostic biomarker and therapeutic target in AML.

## Supplementary information

Supplementary materials and methods

Supplemtary tables

## Data Availability

All data are available in the main text or supplementary materials. Supplementary RNA-seq and ChIRP-seq data are deposited in the Gene Expression Omnibus (GEO) repository under the accession numbers GSE299161 and GSE299541, respectively.
