# Supplementary material for: MALNC: a new mutant NPM1/IDH2R140 and PML-RARA-associated lncRNA with impact on AML cell proliferation, maturation and drug response
Source: Cancer Gene Ther. 2025 Aug 23;32(11):1191–205. doi: 10.1038/s41417-025-00954-0 (PMC12620280; doi:10.1038/s41417-025-00954-0)
Supplement: Supplementary file 1 — Supplementary materials and methods [file 41417_2025_954_MOESM1_ESM.pdf]

Supplementary Materials

MALNC: A new mutant *NPM1/IDH2*^{R140} and *PML-RARA*-associated lncRNA with impact on AML cell proliferation, maturation and drug response

Elisabetta Cozzi *et al.*

Content:

1. Additional material and methods
2. Supplemental figure 1-11
3. Supplemental references

Corresponding authors:

Elisabetta Cozzi
Center of Hematology and Regenerative Medicine, Department of Medicine, Karolinska, Medicinaren 25/Neo, HERM plan 7 / HERM floor 7, 141 83 Huddinge, Sweden.

E-mail: elisabetta.cozzi@ki.se

Sören Lehmann, MD, PhD,
Department of Medical Sciences, Hematology, Uppsala University, Rudbeck Laboratory 751 85 Uppsala, Sweden
and
Center of Hematology and Regenerative Medicine, Department of Medicine, Karolinska, Medicinaren 25/Neo, HERM plan 7 / HERM floor 7, 141 83 Huddinge, Sweden.

E-mail: soren.lehman@medsci.uu.se, soren.lehmann@ki.se

1. Additional Materials and Methods

AML patient cells and healthy donor cells collection

All patient samples in this study were derived from the bone marrow (BM) or peripheral blood (PB) of newly diagnosed AML patients before the start of treatment. Mononuclear cells from the diagnostic samples were isolated by Ficoll-Plaque density-gradient centrifugation (GE Healthcare) before they were vitally frozen. Cells from healthy donors (NBM, normal bone marrow) were aspirated from the BM and enriched for $CD34^+$ cells using magnetic-activated cell sorting (MACS) with positive selections (Miltenyi Biotec). AML patient samples and healthy donor samples were collected after informed consent and in accordance with the Declaration of Helsinki. The study was approved by the regional ethical review board in Stockholm.

Gene expression analyses by RNA-sequencing and differential expression analyses

The initial deep RNA-sequencing and differential expression analysis was performed as described by Qu *et al*(1). RNA from AML patient cells (n=7) and $CD34^+$ NBM cells (n=5) was extracted using TRizol (Life Technologies), followed by the removal of ribosomal RNA by Ribominus Eukaryote kit for RNA-seq (Life Technologies) according to manufacturer's low input protocol. RNA concentrations of pre- and post-Ribominus-treated input RNA were measured using a Bioanalyzer (Agilent). Libraries were prepared with TruSeq RNA Library Preparation Kit v2 (Illumina), equimolarly pooled and quality validated using a TapeStation System (Agilent). In total, 30 ng of Ribominus-treated RNA was used as input material per sample. The libraries were sequenced on six lanes on a HiSeq™ 2000 Sequencing System (Illumina). Deep single-end RNA-sequencing obtained an average of 114M reads for each sample (range: 100M-136M). In total, 1.4 billion reads were generated and 97% of these reads were assigned to input samples. Single-end reads were mapped to the human genome annotation reference GRCh37 (hg19) using TopHat. Then, aligned reads from all 12 samples were combined and a transcriptome assembly was generated using Cufflink and annotated to the GRCh37 genome reference (obtained from Ensemble) using Cuffcompare.

RNA-sequencing from the KAW (AML patients and NBM), ClinSeq-AML, TCGA-LAML (AML patients) cohorts were performed as described (2–5). Differential expression analysis was also performed on a subset of the ClinSeq cohort (n=136) with either very high MALNC expression ($MALNC^{highExp}$, CPM > 5, n=38) or AML patients with complete absence of expression counts ($MALNC^{noExp}$, CPM = 0, n=98) using DESeq2 (v1.26.0) (6,7).

RNA-sequencing of *MALNC*^{KO} NB4 clones was done by extracting total RNA (RNeasy Mini Kit, Qiagen) from three NB4^{KOA}, three NB4^{KOB} and three NB4^{WT} clones. Additionally, one NB4^{KOB} clone and one NB4^{WT} clone were treated in three biological replicates with ATRA (1µM) or vehicle control (DMSO, 0,01%) for 72h and subject to total RNA extraction. RNA from all 15 samples was resuspended in 30 µl of nuclease-free water. Quality control was done by RNA electrophoresis (TapeStation, Agilent Technology Inc, RIN>9). Sequencing libraries were prepared from 500ng total RNA using TruSeq stranded total RNA library preparation kit with ribosomal depletion using RiboZero Gold (Illumina Inc.) and samples were paired-end sequenced (150 cycles in one NovaSeq 6000 SP flowcell). Counts were generated using FeatureCounts and differential expression analysis was performed using DESeq2 (v1.26.0). P-values were determined by the Wald test and adjusted by Benjamini–Hochberg correction.
Gene ontology (GO) and pathway analysis were performed using GOrilla (Gene Ontology enRIchment anaLysis and visuaLizAtion tool) (https://cbl-gorilla.cs.technion.ac.il) (8). Analysis was performed using a target gene list against a background gene list with a p-value cut-off of 0.01. Characteristics of the samples used for RNA-seq are shown in Supplementary Table 28. RNA sequencing data are deposited in the Gene Expression Omnibus (GEO) repository under the accession number GSE299161.

Analysis of MALNC expression in normal hematopoietic cells

MALNC RNA expression was analyzed in bone marrow-derived cell populations from healthy donors at various stages of myeloid maturation, including promyelocytes(CD45+;CD14-;CD13+;CD11b-;CD117+), myelocytes(CD45+; CD14-; CD13+; CD11b+; CD117+), metamyelocytes(CD45+; CD14-; CD13+ CD11b++; CD117+), and monocytes(CD45+;CD14+; CD13+; CD11b+; CD117-). These populations were isolated using magnetic-activated cell sorting (MACS) and fluorescent-activated cell sorting (FACS).

In parallel, human CD34+ hematopoietic stem and progenitor cells were cultured over a 20-day period to assess MALNC expression during granulocytic differentiation. The cells were maintained under sequential cytokine conditions: SCF, FLT3, TPO (50 µg/mL each), and UM171 (70 nM) were used during the first 7 days to preserve stemness and primitive progenitor states. IL3 (25 µg/mL) and GM-CSF (50 µg/mL) were added for the subsequent 5 days to promote early myeloid differentiation, followed by high-dose G-CSF (100 µg/mL) for an additional 5 days to drive granulocytic differentiation. FACS analysis was performed at each time point to evaluate phenotypic changes and lineage progression. Differentiation markers used fat each time point were as follows : 7 days (80% CD34+ CD38-, 15% CD34+ CD38- CD90+ CD45RA-), 12 days(100% CD66b-, 76% CD45RA+, 43% CD34+),

17 days (38% CD66b+ CD45RA-, 42% CD16+, 35% CD34 dim CD45RA+), 20 days (100% CD34-, 51% CD66b+, 57% CD11b+)

Antibodies used to detect cell differentiation stages included 7-Amino-Actinomycin D (7-AAD; Invitrogen, Carlsbad, CA, USA) as a viability dye, along with a panel of antibodies: CD11b (BioLegend, cat. #301404), CD14 (BD Biosciences, cat. #561116), CD19 (BD Biosciences, cat. #561295), CD33 (BD Biosciences, cat. #561160), CD34 (BD Biosciences, cat. #562577), CD38 (BD Biosciences, cat. #562665), CD49d (BD Biosciences, cat. #744751), CD45RA (BD Biosciences, cat. #560674), CD61 (BD Biosciences, cat. #564173), CD90 (BD Biosciences, cat. #561558), CD16 (BD Biosciences, cat. #561304), and CD123 (BioLegend, cat. #306022), CD117 (BD Biosciences, cat. #333233).

For each isolated population of cells , RNA was extracted, and MALNC expression was quantified using qRT-PCR. Full details of qRT-PCR assay sequences are provided in Supplementary Table 1.

Additionally, we retrieved raw data from GEO by accession number GSE98946 (PMID: 28794406). The dataset has stem cells, myeloblasts, promyelocytes, metamyelocytes, and neutrophils based on FACS sorting. FASTQ files were then performed adapter trimming, aligning to the reference genome, and counting using nfcore rnaseq (version 1.4) pipeline.

Primer walk

In order to confirm the entire transcript sequence and to identify lncRNA isoforms, primer walk was performed on HL60 and NB4 cells. For this, predicted exonic regions were used as starting points, based on sequence information from the Functional Annotation of the Mammalian genome (FANTOM) 5 project data (RIKEN, Japan) as well as information from the aligned sequencing reads from the AML cohort of the Clinical Sequencing of Cancer in Sweden (ClinSeq-AML) transcriptome data. Briefly, cDNA was prepared as described for qRT-PCR and amplified by PCR using specific primer pairs and DreamTaq PCR master mix 2X (Thermo Scientific), according to the manufacturer’s protocol. Amplified transcripts were separated on 0.8% agarose-TAE gel, and purified using QIAquick Gel Extraction Kit (Qiagen). DNA recovery was measured using NanoDrop (Thermo Scientific) and amplicons were cloned into pCR™2.1-TOPO® vector (Thermo Scientific). Cloning was done using chemically-competent *Topo10 E.coli* for transformation and QIAprep Spin Miniprep Kit (Qiagen) for plasmid DNA extraction from single colonies. Correct TOPO-TA ligation was confirmed by restriction digest (PmeI/NotI-HF) followed by gel electrophoresis. Plasmids were sequenced by Sanger-sequencing using M13 Reverse primer 5′-CAGGAAACAGCTATGAC-3′ and additional gene internal primer if needed. Then, sequencing results were blasted against the GRChr37 human reference

genome using IGV. Primer sequences used for the primer walk are listed in Supplementary Table 29.

Rapid Amplification of cDNA Ends (5′RACE and 3′RACE)

Transcript start and terminal sites were validated using Rapid Amplification of cDNA Ends (RACE). 3′RACE was done using the 3′ RACE System for Rapid Amplification of cDNA Ends (Thermo Scientific) and FirstChoice™ RLM-RACE Kit (Invitrogen). 5′RACE was done using FirstChoice™ RLM-RACE Kit (Invitrogen). Both 3′ and 5′RACE protocols were done according to the manufacturer's protocols. In short, RNA was extracted from HL60 and NB4 cells as described before and cDNA was synthesized using gene-specific primers. PCR amplification of cDNA was done using DreamTaq PCR Master Mix (Thermo Scientific). The amplicon of the first PCR was used as input (0.1%) for a nested PCR. Nested PCR products were separated by agarose gel, gel extracted and cloned into chemically-competent *Topo10 E.coli* using TOPO TA cloning (pCR™2.1-TOPO™ vector, Invitrogen). Correct TOPO-TA ligation was confirmed by restriction digest (PmeI/NotI-HF) followed by gel electrophoresis and isolated plasmid DNA (Plasmid Midi Kit, Qiagen) was Sanger sequenced as described for primer walk. 3′RACE was performed with both using native RNA or polyadenylated RNA (Yeast poly(A) polymerase, Thermo Scientific). RACE primer sequences are listed in Supplementary Table 30.

RNA secondary structure prediction

The secondary structure of MALNC was determined using representative isoform sequences of MALNC. Secondary RNA structure prediction was performed using mFold web server (http://rna.tbi.univie.ac.at/cgi-bin/RNAWebSuite/RNAfold.cgi, University of Vienna). Fold algorithms were based on minimum free energy (MFE secondary structure) and partition function, avoidance of isolated base pairs and coloration by base-pair probabilities. Isoforms consisted of Ex.1.0-Ex10, Ex1.1-Ex10 and Ex1.2-Ex10, all comprising of all in-between exon except for exon 7, which is mutually exclusive with exon 8-9-10.

***In silico* coding prediction**

In silico coding probability prediction of currently annotated gene *LOC105370601* and the longer *MALNC* gene was determined using CPC2.0 web tool application (Coding Potential Calculator, coding cut-off ≥ 0.5, http://cpc2.gao-lab.org/index.php) and CPAT web tool application (Coding Potential Assessment tool, coding cut-off >0.364, https://wlcb.oit.uci.edu/cpat) (9–11). Coding probability was determined in comparison to well-investigated coding (TNF, POLR2A, GAPDH, AKT1, TBP) and non-coding (HOTAIR, PANDAR, MEG3, XIST, NEAT1, HOTTIP, MALAT1, CRNDE, GAS5, UCA1, CCAT1) genes. Further, i*n silico* coding probability prediction was performed on six major transcript isoforms of MALNC and its full-length transcript using CPAT and CPC.

Polysome fractionation

The non-coding potential of MALNC was further determined by its association with polysomes. Thus, polysome fractionation profiling was carried out to determine the translational capacity of MALNC based on its association with ribosomes and/or its functional implication in translation by interaction with ribosomes. In short, cells ($50\text{-}60\times10^6$ cells/experiment) were washed twice in 1mL PBS supplemented with cycloheximide (100ug/ml, Sigma-Aldrich) and lysed in 425µl lysis buffer (5 mM Tris-HCl (pH 7.5), 2.5 mM MgCl2, 1.5 mM KCl, 1x protease inhibitor cocktail (EDTA-free, Thermo Scientific) and RNAse free ddH2O) supplemented with cycloheximide (5µl, 10ug/ml), DTT (1µl, 1M) and RNase inhibitor (3µl, 40U/µl, Promega). Lysate was vortexed (5sec), supplemented with 100µl of 5% Triton X100 and 5% sodium deoxycholate (1:1) solution and vortexed again (5sec). Lysates were then centrifuged (10 min, 14,000 x *g*, 4°C) and the supernatant (cytoplasmic lysate) was transferred to a fresh tube. Each lysate was spiked with luciferase RNA (uncapped exogenous RNA used as non-translated RNA control), then cell lysates were pipetted onto a linear sucrose gradient (15-50%) for ultracentrifugation (4°C). The gradient was analyzed by measuring RNA absorbance (254nm) and fractionated into 27 polysome fractions. For analysis, fractions were pooled into 13 fractions, spiked with an equal amount of artificial XENO RNA (qRT-PCR normalization control, TaqMan® Cells-to-CT™ Control Kit, Ambion), followed by RNA extraction using Trizol (Thermo Scientific) and qRT-PCR analysis (TaqMan® Gene Expression Cells-to-CT™ Kit, Ambion). Expression was normalized to XENO RNA spike-in and relative abundance of MALNC, luciferase RNA (uncapped exogenous RNA used as non-translated RNA control) and ACTB RNA (well-transcribed RNA control) were determined per polysome fraction over total RNA detected.

DNase-sequencing and ChIP-sequencing data

DNase I hypersensitive sites (DHSs) sequencing (DNase-seq) data and chromatin immunoprecipitation sequencing (ChIP-seq) data were retrieved from the public depository of the Encyclopedia of DNA Elements Consortium (ENCODE Project Portal, https://www.encodeproject.org/).

DNase-seq data was obtained from HL60, NB4 and K562 cell lines and primary $CD34^+$ cells from healthy donors. RNA polymerase II ChIP-seq (POLR2A-seq) was obtained from HL60 and K562 cell lines. Histone ChIP-seq on histone 3 lysine 4 trimethylation (H3K4me3) and histone 3 lysine 27 acetylation (H3K27ac) was obtained from ATRA-treated and control-treated HL60 cells, NB4 and K562 cell lines. Transcription factor (TF) ChIP-seq on c-Myc and Max was obtained from NB4 and K562 and TF ChIP-seq on REST and PU.1 (SPI1) was obtained from HL60 and K562 Samples are listed in Supplementary Table 31.

Binding motifs of MALNC

MALNC promoter regions were investigated for binding motif sites by using the *´Find Motif´* function in Integrative Genome Viewer (IGV) and known consensus sequences for TATA box motif as well as for common elements and transcription factors including HNF4, AP1, OCT1, CHOP-C/EBPα, and MECOM (EVI1) motifs in the vicinity of transcriptional start sites.

Conservation of lncRNA MALNC

The evolutionary conservation of lncRNA MALNC was analyzed by homology and sequence alignment search using Basic Local Alignment Search Tool (nucleotide BLAST, NCBI) and Evolutionary Conserved Regions bowser (http://ecrbrowser.dcode.org), where multiple alignments between different species reveal evolutionarily conserved regions (ECRs). ECRs were defined as a minimal length of 50bps and a minimum sequence identity of 70%.

Statistical testing and data analysis of clinical correlations

The ClinSeq-AML and TCGA-LAML cohorts were used to identify significant correlations with clinical parameters, such as with genetic and cytogenetic AML risk categories, AML subgroup and mutational status. P-value was determined by Pearson correlation for continuous variables (age at diagnosis, blood values), Student´s t-test or Wilcoxon rank-sum test for pairwise comparisons (binary parameters such as mutation status) and Kruskal–Wallis test by ranks for multi-class parameters (etiology, cytogenetic and genetic risk groups).Dunn´s test with Bonferroni correction was done for multiple testing (globally or by analysis) for comparisons among groups. The correlation of lncRNA expression with overall survival (OS, from diagnosis to last status) was analyzed using Kaplan-Meier curves on dichotomized expression data based on median gene expression. CPM > median was defined as high expression and CPM < median was defined as low expression. Overall survival data was censored for allogeneic hematopoietic stem cell transplantation (allo-HSC) and the p-value of dichotomized data was determined by the Mantel–Cox log-rank test. Uni-and multivariate regression analysis was done to evaluate MALNC independent prognostic value by including markers with known prognostic impact.

Drug sensitivity and resistance testing and validation

High-throughput drug sensitivity and resistance testing (DSRT) was carried out as previously described (9). In short, cells were seeded at 2,000-5,000 cells/well onto 8,384 well drug library plates (acoustically dispensed drugs of FO5A drug library comprising 525 unique drugs: FDA approved 30%, investigative 53% and developmental 17%) in a 5-point 10,000-fold concentration range. A selective Drug Sensitivity Score (sDSS) was calculated for each drug by subtracting the DSS of the control cells (MALNC WT cells) from the DSS of the sample of interest (MALNC KO cells), providing a quantitative measure of differential drug response.

For drug sensitivity validation testing, cells were seeded at 18,000 cells/well onto a 96-well plate and treated with 10-11-point serial diluted drug compounds or vehicle control. After incubation of 72h (37 °C and 5 % CO_2), viability after treatment was measured by CellTiter-Glo® 2.0 Cell Viability Assay (Promega) using a Synergy HTX plate reader.

Cell cycle analysis

Cell cycle analysis was done using propidium iodide (PI) Flow Cytometry Kit (Abcam). Cells were washed once in 1x PBS, fixed in 66% ice-cold ethanol and stored at -4°C for up to 48h. Thereafter, cells were resuspended by pipetting, washed again in PBS, resuspended in 200µl propidium iodine solution supplemented with RNase. Cells were then incubated in the dark (37°C, 30 min). After incubation, cells were placed on ice and analyzed by flow cytometry (BC CytoFLEX Flow Cytometer). Data was evaluated by FlowJoTM v10.8.0.

Apoptosis analysis

Cell apoptosis was evaluated using eBioscience™ Annexin V-FITC Apoptosis Detection Kit (Thermo Scientific). In short, cells were washed in PBS, resuspended in 200µl binding buffer and incubated with Annexin V-FITC for 10 minutes at room temperature. Cells were washed again in binding buffer and then resuspended in binding buffer supplemented with propidium iodine (20µg/mL). Cells were immediately analyzed by flow cytometry (BC CytoFLEX Flow Cytometer) and data was evaluated by FlowJo™ v10.8.0.

Code availability

This paper doesn´t include original codes. Codes used for bioinformatic and statistical analysis are available upon request from the corresponding author.

2. Supplemental Figures

Figure S1

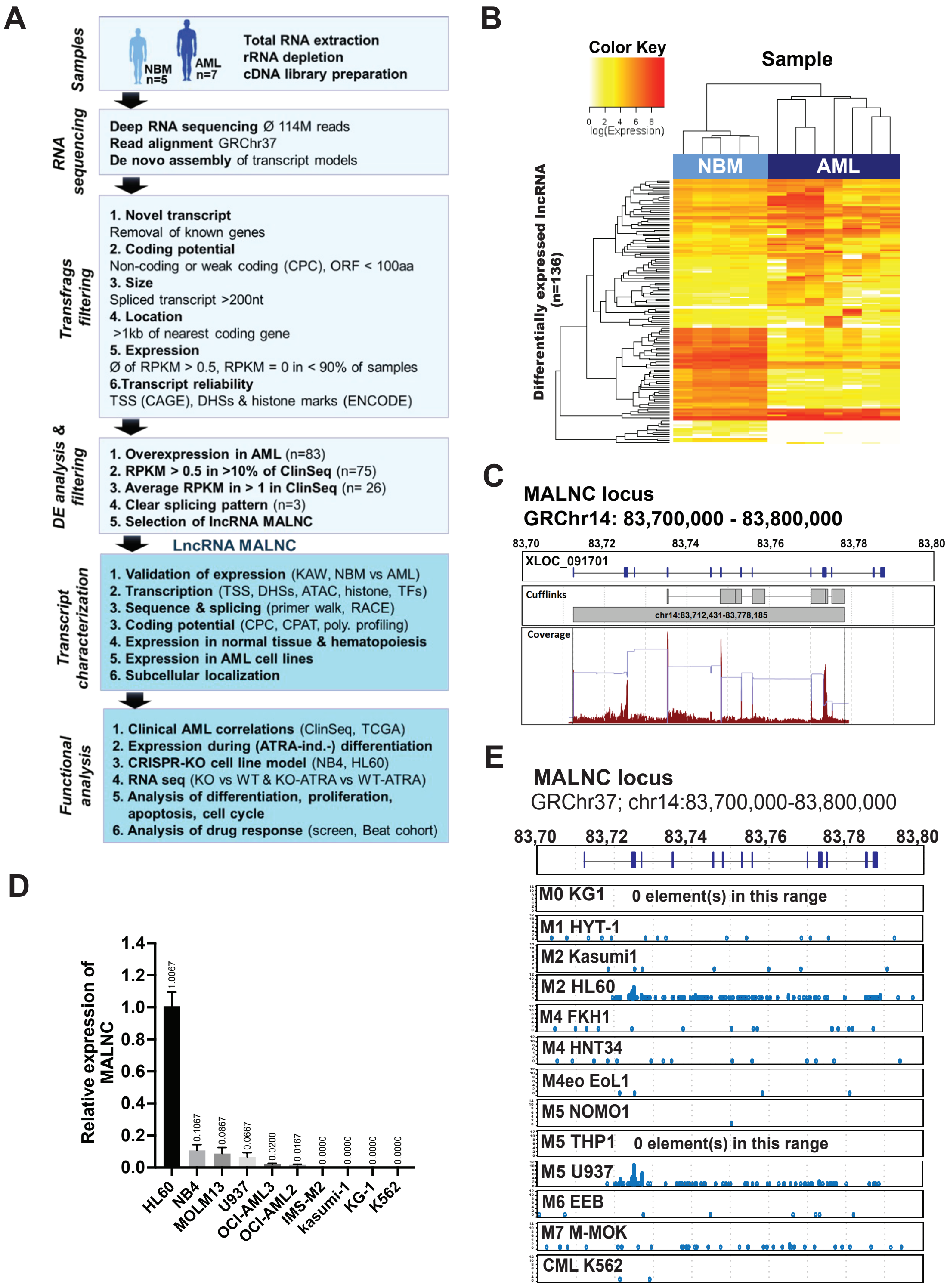

Fig. S1. Discovery, Characterization, and Expression Analysis of MALNC in Leukemic Cell Lines. (A) Schematic overview of lncRNA candidate discovery, filtering and selection and downstream structural validation and functional investigation of lncRNA MALNC. **(B)** Hierarchical clustering of 136 differentially expressed lincRNA genes. Subtype sample marked with column color indicating NBM in light blue and AML in dark blue. **(C)** *(Top)* Exonic structure of *MALNC* as identified in this study. *(Middle)* Initially predicted genomic loci and exon structure of *MALNC* on chromosome 14 by Cufflinks spanning 65,754bp (chr14:14:83712431-83778185, GRChr37). *(Bottom)* Histogram of summed coverage of read alignment in all ClinSeq AML samples (n=325) shown as nucleotide coverage (red bars) and gap coverage (blue line) in the predicted locus (QMAP ≥20 reads). **(D)** Relative RNA expression of lncRNA MALNC (qRT-PCR) in 12 different leukemic cell lines. Data is shown as means ± SEM from three biological experiments relative to HL60 (most abundant expression) and normalized towards endogenous control gene *TBP*. **(E)** Expression of MALNC in 12 different AML cell lines (sorted by FAB classification M0 to M7) and CML cell line K562. Data is shown as FANTOM5 CAGE tag starting sites (CTSS) on the genomic locus of *MALNC* (chr:14:83,700,000-83,800,000, GRCh37). Abbreviations: AML, acute myeloid leukemia; NBM, normal bone marrow cells ($CD34^+$); rRNA, ribosomal RNA; cDNA, complementary DNA; CPC, Coding Potential Calculator; CPAT, Coding Potential Assessing Tool; ORF, open reading frame; TSS, transcription start site; RACE, rapid amplification of cDNA ends; DE analysis, differential expression analysis; CAGE, FANTOM CAGE-assisted transcriptome data.

Figure S2

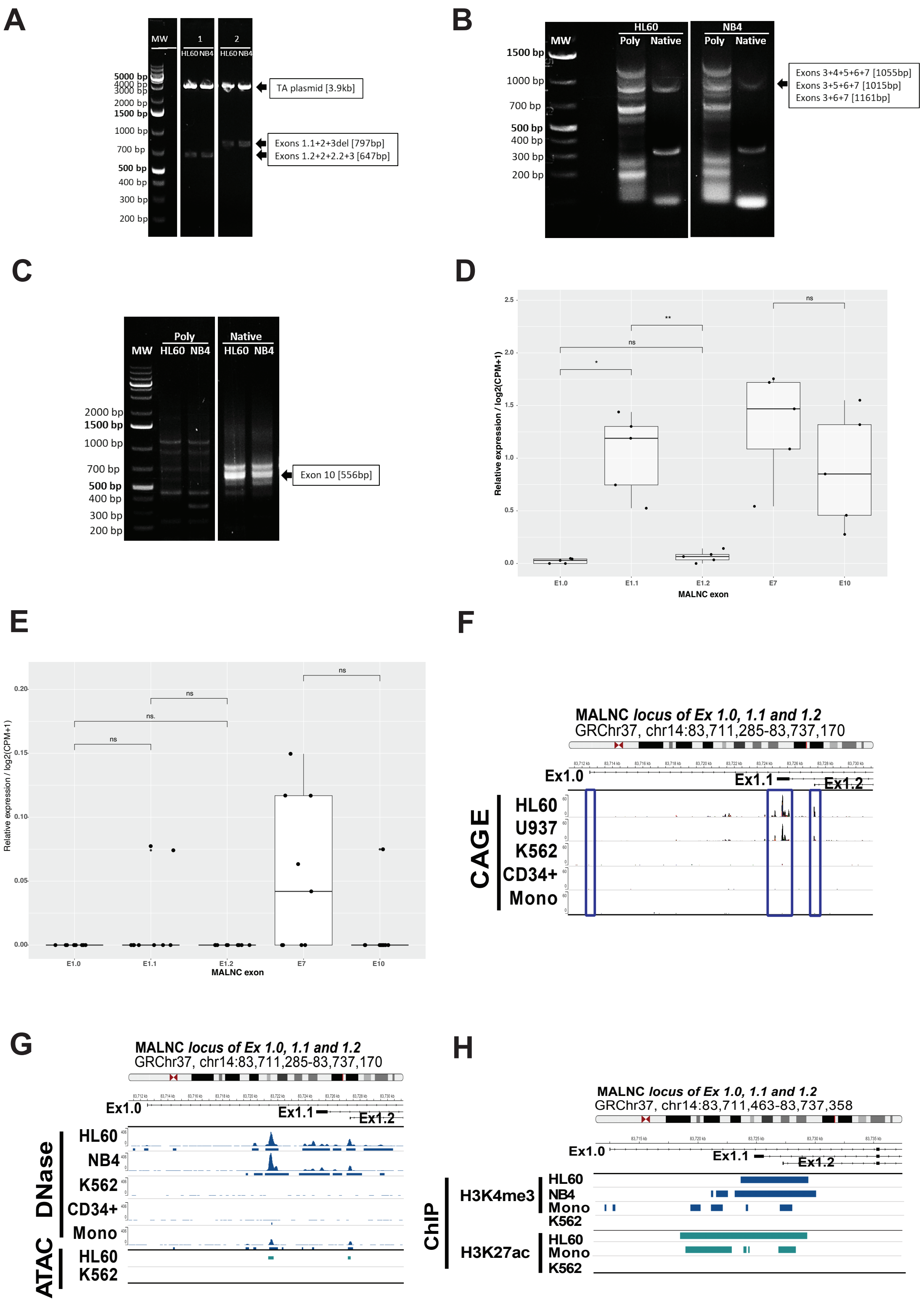

A
MW
1
2
HL60 NB4
HL60 NB4
5000 bp
4000 bp
3000 bp
2000 bp
1500 bp
1000 bp
700 bp
500 bp
400 bp
300 bp
200 bp
TA plasmid [3.9kb]
Exons 1.1+2+3del [797bp]
Exons 1.2+2+2.2+3 [647bp]
B
HL60
NB4
MW
Poly
Native
Poly
Native
1500 bp
1000 bp
700 bp
500 bp
400 bp
300 bp
200 bp
Exons 3+4+5+6+7 [1055bp]
Exons 3+5+6+7 [1015bp]
Exons 3+6+7 [1161bp]
C
Poly
Native
MW
HL60
NB4
HL60
NB4
2000 bp
1500 bp
1000 bp
700 bp
500 bp
400 bp
300 bp
200 bp
Exon 10 [556bp]
D
Relative expression / log2(CPM+1)
MALNC exon
E1.0
E1.1
E1.2
E7
E10
ns
**
ns
*
E
Relative expression / log2(CPM+1)
MALNC exon
E1.0
E1.1
E1.2
E7
E10
ns
ns
ns.
ns
F
MALNC locus of Ex 1.0, 1.1 and 1.2
GRChr37, chr14:83,711,285-83,737,170
Ex1.0
Ex1.1
Ex1.2
CAGE
HL60
U937
K562
CD34+
Mono
G
MALNC locus of Ex 1.0, 1.1 and 1.2
GRChr37, chr14:83,711,285-83,737,170
Ex1.0
Ex1.1
Ex1.2
DNase
HL60
NB4
K562
CD34+
Mono
ATAC
HL60
K562
H
MALNC locus of Ex 1.0, 1.1 and 1.2
GRChr37, chr14:83,711,463-83,737,358
Ex1.0
Ex1.1
Ex1.2
ChIP
H3K4me3
HL60
NB4
Mono
K562
H3K27ac
HL60
Mono
K562

Fig. S2. MALNC Transcription Start and Termination Sites, Chromatin Accessibility, Regulatory Elements, and Expression Modulation. (A) Identified transcription start exons (exon 1.1 and exon 1.2) of MALNC in HL60 and NB4 cell line by 5´RLM-RACE using primer GSP7 and GSP8. Shown is the gel electrophoresis of nested PCR products after cloning into TOPO-TA vector and PmeI/NotI double restriction digest. The undigested plasmid was Sanger sequenced and sequence confirmed. **(B, C)** Identified transcription termination exon 7 and exon 10 of MALNC by 3´RLM-RACE using primer GSP1 and GSP2 in HL60 and NB4 cells. Termination exons were identified both using polyadenylated (Poly) and untreated (Native) RNA. **(D)** Isoform usage as indicated by expression of MALNC by transcription start exons (exon 1.0, exon 1.1 and exon 1.2) and transcription termination exons (exon 7 and exon 10) among APL patients from KAW cohort (n=5). Data shown as read counts log2(CPM+1) using box and whisker dot plots with interquartile range (IQR). **(E)** Isoform usage as indicated by expression of MALNC by transcription start exons (exon 1.0, exon 1.1 and exon 1.2) and transcription termination exons (exon 7 and exon 10) among healty donor promylocytes from public available data (n = 8; GSE98310). Data shown as read counts log2(CPM+1) using box and whisker dot plots with interquartile range (IQR).**(F)** Transcription start sites (TSS, CAGE-seq) in transcription start site of *MALNC* (chr14:83,711,285-83,737,170, GRCh37) for cell lines HL60, U937 and K562, $CD34^+$ and monocytes. The locus is zoomed on all three start exons of *MALNC* (Ex. 1.0, Ex 1.1 and Ex 1.2). Data retrieved from the FANTOM5 project. Data scaled by the group was visualized using IGV. **(G)** DNase hypersensitivity sites (DHS, DNase-seq) and chromatin accessibility (ATAC-seq) in the transcription start site of *MALNC* (chr14:83,711,285-83,737,170, GRCh37) for cell lines HL60, NB4 and K562 and healthy $CD34^+$ and monocytes. Data retrieved from ENCODE (DNaseI Hypersensitivity by Digital DNaseI from ENCODE/University of Washington, broad peak and bigwig files.) **(H)** Histone modifications H3K4me3 and H3K27ac (ChIP-seq) in transcription start site of *MALNC* (chr14:83,711,463-83,737,358, GRCh37) for cell lines HL60, NB4 and K562 and healthy monocytes. Data retrieved from ENCODE (GSE93994 and GSE136759). P-values were determined by two way ANOVA followed by pairwise comparison testing (2D-E): ns- not significant, *p-value < 0.05, **p-value < 0.01, ***p-value < 0.001.

Figure S3

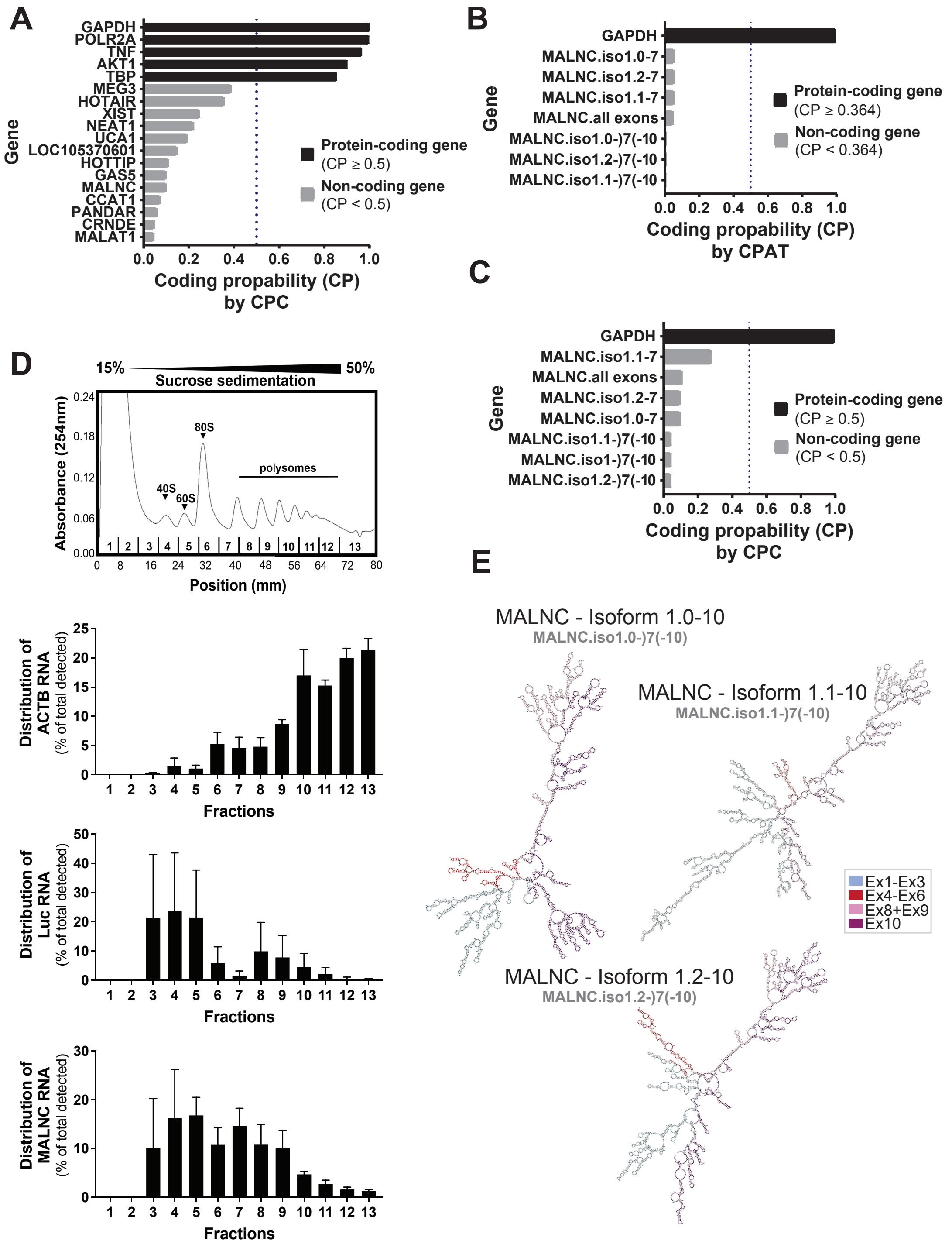

A
GAPDH
POLR2A
TNF
AKT1
TBP
MEG3
HOTAIR
XIST
NEAT1
UCA1
LOC105370601
HOTTIP
GAS5
MALNC
CCAT1
PANDAR
CRNDE
MALAT1
Gene
Protein-coding gene (CP ≥ 0.5)
Non-coding gene (CP < 0.5)
0.0 0.2 0.4 0.6 0.8 1.0
Coding propability (CP) by CPC
B
GAPDH
MALNC.iso1.0-7
MALNC.iso1.2-7
MALNC.iso1.1-7
MALNC.all exons
MALNC.iso1.0-)7(-10
MALNC.iso1.2-)7(-10
MALNC.iso1.1-)7(-10
Gene
Protein-coding gene (CP ≥ 0.364)
Non-coding gene (CP < 0.364)
0.0 0.2 0.4 0.6 0.8 1.0
Coding propability (CP) by CPAT
C
GAPDH
MALNC.iso1.1-7
MALNC.all exons
MALNC.iso1.2-7
MALNC.iso1.0-7
MALNC.iso1.1-)7(-10
MALNC.iso1-)7(-10
MALNC.iso1.2-)7(-10
Gene
Protein-coding gene (CP ≥ 0.5)
Non-coding gene (CP < 0.5)
0.0 0.2 0.4 0.6 0.8 1.0
Coding propability (CP) by CPC
D
15%
50%
Sucrose sedimentation
Absorbance (254nm)
40S
60S
80S
polysomes
Position (mm)
Distribution of ACTB RNA (% of total detected)
Fractions
Distribution of Luc RNA (% of total detected)
Fractions
Distribution of MALNC RNA (% of total detected)
Fractions
E
MALNC - Isoform 1.0-10
MALNC.iso1.0-)7(-10)
MALNC - Isoform 1.1-10
MALNC.iso1.1-)7(-10)
Ex1-Ex3
Ex4-Ex6
Ex8+Ex9
Ex10
MALNC - Isoform 1.2-10
MALNC.iso1.2-)7(-10)

Fig.S3. Coding Probability, Polysome Association, and Secondary Structure Analysis of MALNC. (A) *In silico* coding probability prediction of currently annotated gene *LOC105370601* and the longer *MALNC* gene in comparison to well-investigated coding genes (TNF, POLR2A, GAPDH, AKT1, TBP) and non-coding (HOTAIR, PANDAR, MEG3, XIST, NEAT1, HOTTIP, MALAT1, CRNDE, GAS5, UCA1, CCAT1)_The coding probability was determined by the CPC2.0 web tool application (Coding Potential Calculator, coding cut-off ≥ 0.5). **(B, C)** *In silico* coding probability prediction of six major transcript isoforms of MALNC and its full-length transcript using CPAT and CPC. Coding probability was determined by the CPAT web tool application (Coding Potential Assessment tool, coding cut-off >0.364) and by the CPC2.0 web tool application (Coding Potential Calculator, coding cut-off ≥ 0.5). **(D)** Non-coding potential of MALNC was determined by its association with polysomes. Cell lysates from NB4 cells were analyzed by 15-50% sucrose density gradient fractionation. RNA was extracted from each gradient fraction and expression of *ACTB*, Luciferase RNA and MALNC was determined by qRT-PCR. *(Top)* Sucrose gradient absorbance profile (254nm) from NB4 cells indicating the localization of free RNA, ribosomal subunits (40S and 60S), monosomes (80S) and polysomes along the sampled sucrose gradient fractions (1-13). One representative absorbance profile is shown. *(Bottom)* Bar charts representing the distribution of co-sedimented beta-actin (*ACTB*), luciferase (Luc) and MALNC RNA transcripts in NB4 from each sucrose gradient fraction as a percent of total RNA detected by qRT-PCR. Data is shown as mean with SEM from 4 fractionations and was normalized to spiked-in XENO RNA. **(E)** Secondary structure of MALNC lncRNA was investigated by RNAfold webserver and three isoforms were selected to represent MALNC secondary structure folding based on minimal free energy prediction (MFE secondary structure). Isoforms consisted of Ex.1.0-Ex10, Ex1.1-Ex10 and Ex1.2-Ex10, all comprising of all in-between exons except for exon 7, which is mutually exclusive with exon 8-9-10.

Figure S4

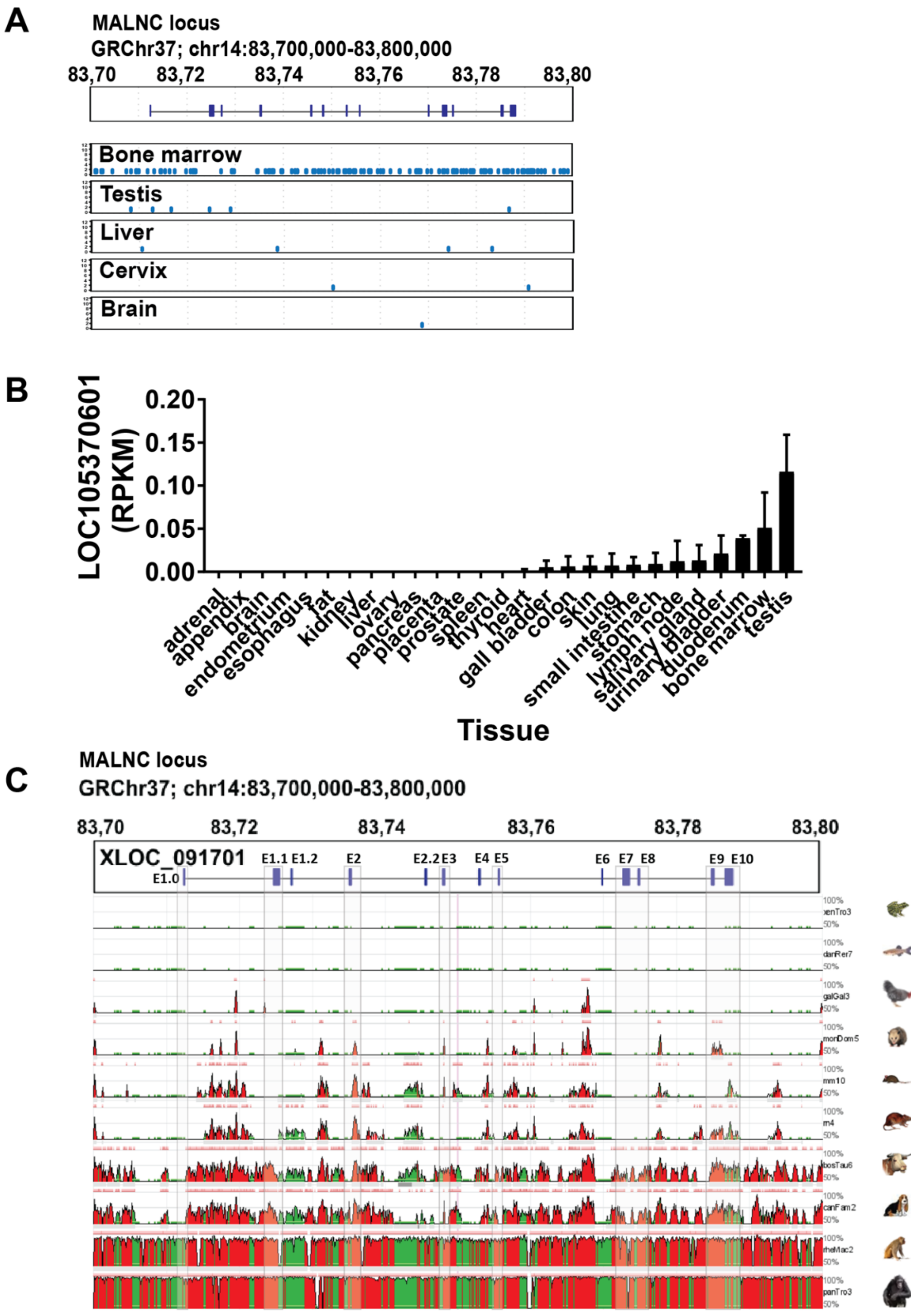
A
MALNC locus
GRChr37; chr14:83,700,000-83,800,000
83,70
83,72
83,74
83,76
83,78
83,80
Bone marrow
Testis
Liver
Cervix
Brain
B
LOC105370601 (RPKM)
0.20
0.15
0.10
0.05
0.00
adrenal
appendix
brain
endometrium
esophagus
fat
kidney
liver
ovary
pancreas
placenta
prostate
spleen
thyroid
heart
gall bladder
colon
skin
lung
small intestine
stomach
lymph node
salivary gland
urinary bladder
duodenum
bone marrow
testis
Tissue
C
MALNC locus
GRChr37; chr14:83,700,000-83,800,000
83,70
83,72
83,74
83,76
83,78
83,80
XLOC_091701
E1.0
E1.1
E1.2
E2
E2.2
E3
E4
E5
E6
E7
E8
E9
E10

Fig. S4. MALNC is a non-coding intergenic RNA specifically expressed in bone marrow. **(A)** Expression of MALNC in different normal tissue types using FANTOM5 CAGE tag starting sites (CTSS). Data is shown as 5′-end of the mapped CAGE reads counted at a single base pair resolution (CAGE tag starting sites, CTSS signal) on the genomic locus of *MALNC*(chr:14:83,700,000-83,800,000, GRCh37), which represents TSS activities in the sample. **(B)** RNA expression of lncRNA *LOC105370601* (RPKM, Total RNA-seq) in 27 different normal tissue types (n=2-7, HPA RNA-seq normal tissues BioProject: PRJEB4337). **(C)** Screenshot of ECR browser indicating the evolutionary conserved regions (sequence similarity, %) within the *MALNC* locus (chr14:83,700,00-83,800,00) between the human (GRCh37) and different vertebrate genomes (frog (xenTro3), zebrafish (danRer7), chicken (galGal3), opossum (monDom5), mouse (mm10), rat (rn4), cow (bosTau6), dog (canFam2), rhesus macaque (rheMac2) and chimpanzee (panTro3)). Shown are evolutionarily conserved regions (ECRs) with a minimum length of 50bp and at least 70% sequence identity, where peaks indicate genomic regions with high sequence similarities.

Figure S5

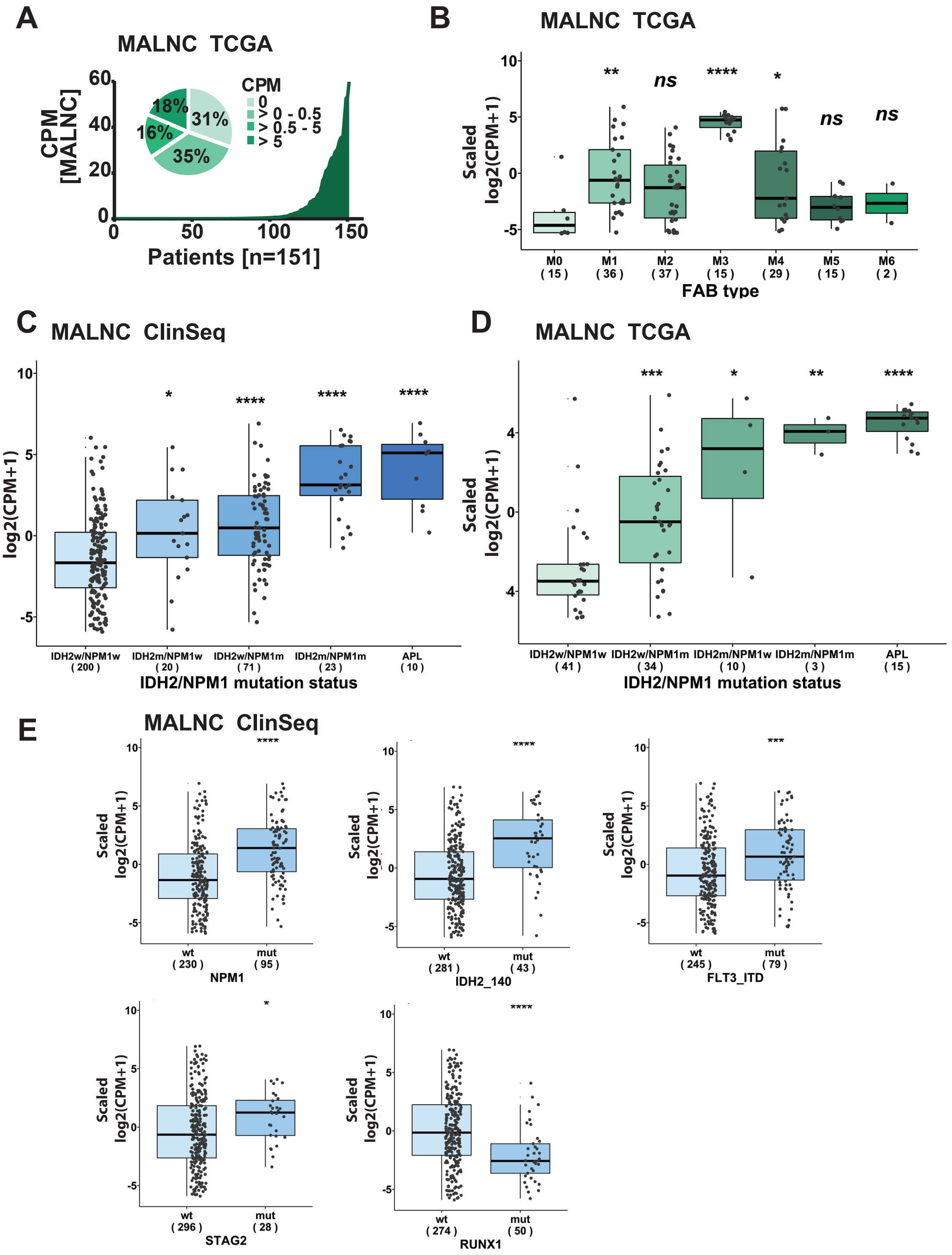
A
MALNC TCGA
CPM [MALNC]
Patients [n=151]
CPM
0
> 0 - 0.5
> 0.5 - 5
> 5
31%
35%
16%
18%
B
MALNC TCGA
Scaled log2(CPM+1)
FAB type
M0 (15)
M1 (36)
M2 (37)
M3 (15)
M4 (29)
M5 (15)
M6 (2)
C
MALNC ClinSeq
IDH2w/NPM1w (200)
IDH2m/NPM1w (20)
IDH2w/NPM1m (71)
IDH2m/NPM1m (23)
APL (10)
IDH2/NPM1 mutation status
D
MALNC TCGA
IDH2w/NPM1w (41)
IDH2w/NPM1m (34)
IDH2m/NPM1w (10)
IDH2m/NPM1m (3)
APL (15)
IDH2/NPM1 mutation status
E
MALNC ClinSeq
wt (230) mut (95) NPM1
wt (281) mut (43) IDH2_140
wt (245) mut (79) FLT3_ITD
wt (296) mut (28) STAG2
wt (274) mut (50) RUNX1

Fig. S5. MALNC associates with APL, promyelocytes and IDH2^{R140}/NPM1-mutated AML. (A) Bar chart presenting the distribution of MALNC CPM counts among AML patients in the TCGA-LAML cohort (n=151) and pie chart presenting the percentage of AML patients classified as MALNC non-expressors (CPM=0 and CPM >0 -0.5) and MALNC expressors (CPM > 0.5 – 5 and CPM > 5). (**B**) Expression of MALNC among AML FAB groups. Data from TCGA-LAML cohort. Normalized counts (log2(CPM+1)) were scaled and shown as box and whisker dot plots with interquartile range (IQR). The sample number is indicated in parentheses (n). P-value determined by Kruskal–Wallis test and then followed by Dunn´s pairwise comparison test. **(C, D)** Distribution of MALNC expression in ClinSeq-AML and TCGA-LAML patients with APL, *NPM1/IDH2*^{R140} wild-type AML (IDH2w/NPM1w), *NPM1* wild-type and mutated *IDH2*^{R140} (IDH2m/NPM1w), mutated *NPM1*, wild-type *IDH2*^{R140} (IDH2w/NPM1m) AML and co-occurring *NPM1/IDH2*^{R140} mutation (IDH2m/NPM1m). Normalized counts (log2(CPM+1)) were scaled and shown as box and whisker dot plots with interquartile range (IQR). The sample number is indicated in parentheses (n). P-value determined by Kruskal–Wallis test and then followed by Dunn´s pairwise comparison test. **(E)** Expression of MALNC among *NPM1*, *IDH2*^{R140}, *FLT3_ITD*, *STAG1* and *RUNX1* mutational status of AML patients. Normalized counts (log2(CPM+1)) were scaled and shown as box and whisker dot plots with interquartile range (IQR). The sample number is indicated in parentheses (n). P-value determined by Wilcoxon rank-sum test for pairwise comparisons.

Figure S6

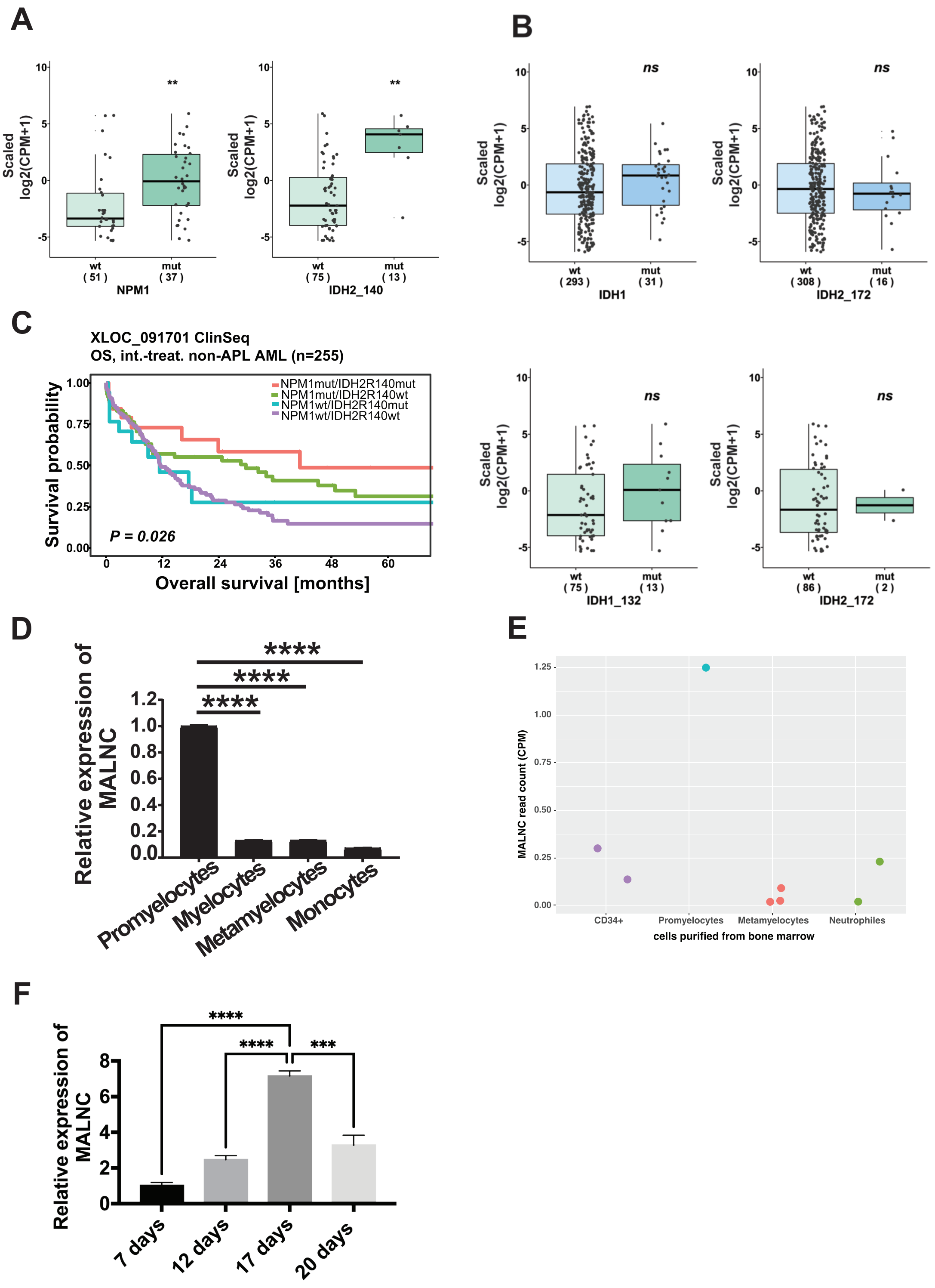
A
Scaled log2(CPM+1)
**
wt (51)
mut (37)
NPM1
wt (75)
mut (13)
IDH2_140
B
ns
wt (293)
mut (31)
IDH1
wt (308)
mut (16)
IDH2_172
wt (75)
mut (13)
IDH1_132
wt (86)
mut (2)
IDH2_172
C
XLOC_091701 ClinSeq
OS, int.-treat. non-APL AML (n=255)
NPM1mut/IDH2R140mut
NPM1mut/IDH2R140wt
NPM1wt/IDH2R140mut
NPM1wt/IDH2R140wt
Survival probability
P = 0.026
Overall survival [months]
D
Relative expression of MALNC
****
Promyelocytes
Myelocytes
Metamyelocytes
Monocytes
E
MALNC read count (CPM)
CD34+
Promyelocytes
Metamyelocytes
Neutrophiles
cells purified from bone marrow
F
Relative expression of MALNC
****
***
7 days
12 days
17 days
20 days

Fig. S6. MALNC associates with APL, promyelocytes and IDH2^{R140}/NPM1-mutated AML. (A) Expression of MALNC among *NPM1* and *IDH2^{R140}* mutated AML patients in the TCGA-LAML cohort. Normalized counts (log2(CPM+1)) were scaled and shown as box and whisker dot plots with interquartile range (IQR). The sample number is indicated in parentheses (n). P-value determined by Wilcoxon rank-sum test for pairwise comparisons. **(B)** Expression of MALNC among *IDH1^{R132}* and *IDH2^{R172}* mutated AML patients in *(top)* ClinSeq-AML and *(bottom)* TCGA-LAML cohort. Normalized counts (log2(CPM+1)) were scaled and shown as box and whisker dot plots with interquartile range (IQR). The sample number is indicated in parentheses (n).. P-value determined by Wilcoxon rank-sum test for pairwise comparisons. **(C)** Kaplan-Meier survival curves showing overall survival (OS) of AML patients stratified by IDH140 and NPM1 mutational status. Data is shown in the ClinSeq-AML cohort of intensively treated patients, excluding APL patients (n=255). P-value determined by log-rank test. Data was censored for allogenic stem cell transplantation (HSCT). **(D)** Relative RNA expression of lncRNA MALNC (RT-qPCR) during normal hematopoiesis using FACS-sorted hematopoietic precursor and progenitor cells (promyelocytes, myelocytes, metamyelocytes and monocytes). Data shown as means ± SEM from three technical replicates of one biological experiment, and relative to promyelocytes (most abundant expression) and normalized towards endogenous control gene *TBP*. P-values were determined by Students t-tests: ns- not significant, *p-value < 0.05, **p-value < 0.01, ***p-value < 0.001, ****p-value < 0.0001. (**E)** Dot plot showing MALNC expression represented as read counts (CPM) in stem cells, promyelocytes, metamyelocytes, and neutrophils. Data were retrieved from GSE98946 (PMID: 28794406**). (F)** MALNC RNA expression (RT-qPCR) during CD34+ normal cell differentiation.CD34+ cells were cultured for 20 days under sequential cytokine conditions. Four-time points were collected and FACS analyzed: 7 days (80% CD34+ CD38-, 15% CD34+ CD38- CD90+ CD45RA-),12 days(100% CD66b-, 76% CD45RA+, 43% CD34+), 17 days (38% CD66b+ CD45RA-, 42% CD16+, 35% CD34 dim CD45RA+), 20 days (100% CD34-, 51% CD66b+, 57% CD11b+). Data is shown as means ± SD from three biological experiments relative to 7 days of cell differentiation and normalized towards endogenous control gene *TBP.*

Figure S7

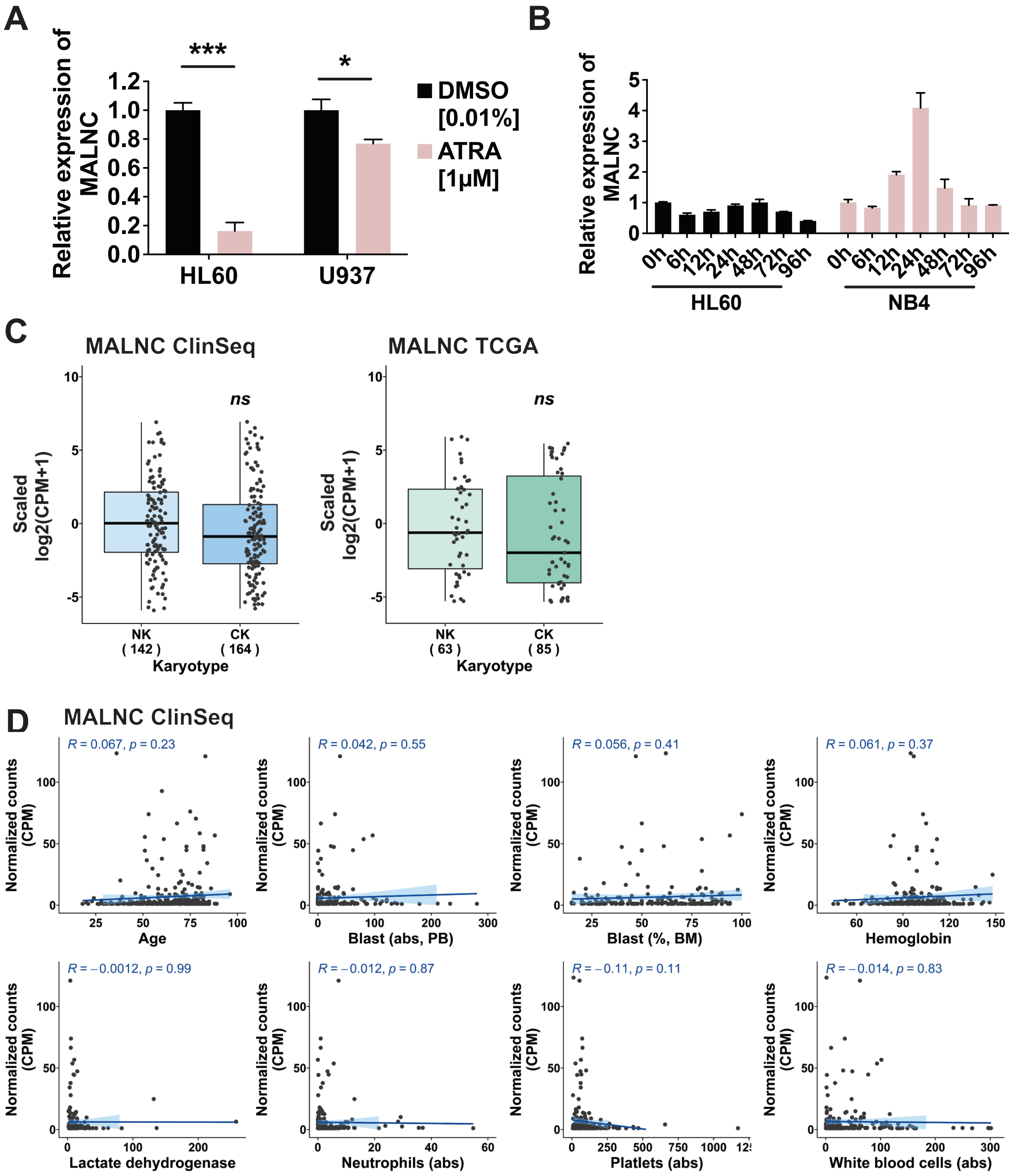
A
Relative expression of MALNC
***
*
DMSO [0.01%]
ATRA [1μM]
HL60
U937
B
Relative expression of MALNC
0h 6h 12h 24h 48h 72h 96h
HL60
NB4
C
MALNC ClinSeq
MALNC TCGA
ns
Scaled log2(CPM+1)
NK (142)
CK (164)
NK (63)
CK (85)
Karyotype
D
MALNC ClinSeq
R = 0.067, p = 0.23
R = 0.042, p = 0.55
R = 0.056, p = 0.41
R = 0.061, p = 0.37
Normalized counts (CPM)
Age
Blast (abs, PB)
Blast (%, BM)
Hemoglobin
R = −0.0012, p = 0.99
R = −0.012, p = 0.87
R = −0.11, p = 0.11
R = −0.014, p = 0.83
Lactate dehydrogenase
Neutrophils (abs)
Platlets (abs)
White blood cells (abs)

Fig. S7. MALNC decreases during ATRA-induced myeloid differentiation. (A) Relative expression of MALNC (qRT-PCR) in HL60 and U937 cells during ATRA-induced differentiation (10µM ATRA, 72h). Data is shown as means ± SEM from three biological experiments relative to vehicle control treatment (DMSO, 0.01%) and normalized towards endogenous control gene *TBP*. **(B)** Relative expression of MALNC (qRT-PCR) in HL60 and NB4 cells during Vitamin D3-induced differentiation (100nM VitD3, 96h). Data is shown as means ± SEM from three biological experiments relative to vehicle control treatment (EtOH, 0.001%) and normalized towards endogenous control gene *TBP*. **(C)** Distribution of MALNC expression stratified by karyotype (normal karyotype- NK; complex karyotype- CK). Data from ClinSeq-AML and TCGA-LAML cohorts. Normalized counts (log2(CPM+1)) were scaled and shown as box and whisker dot plots with interquartile range (IQR). The sample number is indicated in parentheses (n). P-value determined by Wilcoxon rank-sum test for pairwise comparisons. **(D)** Pearson correlation of MALNC expression to numerical clinical parameters including age, blast percentage (PB) and blast count (BM), hemoglobin level, lactate dehydrogenase level, neutrophils, platelets and white blood cell counts). Data from the ClinSeq-AML cohort using CPM. Black dots represent outliers; grey data points represent all data. The sample number is indicated in parentheses (n).

Figure S8

A

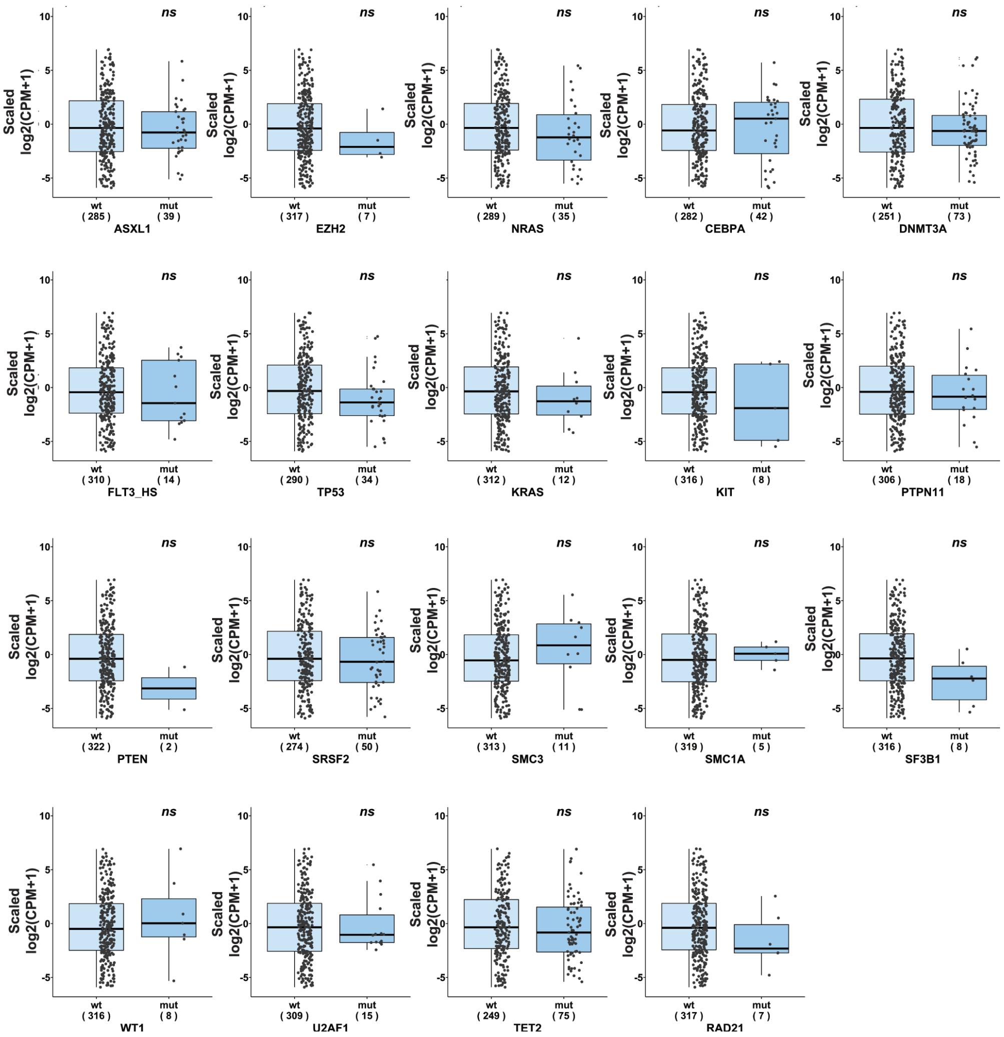
Scaled log2(CPM+1)
ns
wt (285) mut (39) ASXL1
wt (317) mut (7) EZH2
wt (289) mut (35) NRAS
wt (282) mut (42) CEBPA
wt (251) mut (73) DNMT3A
wt (310) mut (14) FLT3_HS
wt (290) mut (34) TP53
wt (312) mut (12) KRAS
wt (316) mut (8) KIT
wt (306) mut (18) PTPN11
wt (322) mut (2) PTEN
wt (274) mut (50) SRSF2
wt (313) mut (11) SMC3
wt (319) mut (5) SMC1A
wt (316) mut (8) SF3B1
wt (316) mut (8) WT1
wt (309) mut (15) U2AF1
wt (249) mut (75) TET2
wt (317) mut (7) RAD21

B

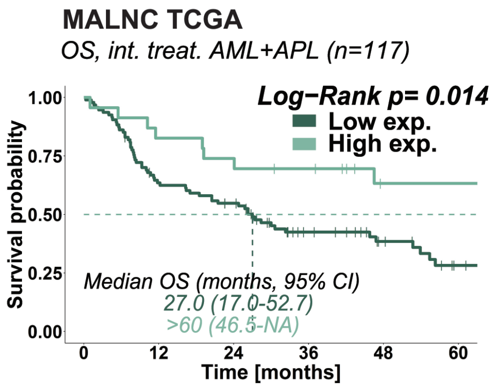
MALNC TCGA
OS, int. treat. AML+APL (n=117)
Log−Rank p= 0.014
Low exp.
High exp.
Survival probability
Median OS (months, 95% CI)
27.0 (17.0-52.7)
>60 (46.5-NA)
Time [months]

Fig. S8. MALNC associates with better OS in AML patients. (A) Box and Whisker plots of MALNC expression stratified by mutational status of commonly mutated genes in AML. Data from ClinSeq cohort. Normalized counts (log2(CPM+1)) were scaled and shown as box and whisker dot plots with interquartile range (IQR). The sample number is indicated in parentheses (n). P-value determined by Wilcoxon rank-sum test for pairwise comparisons. **(B)** Kaplan-Meier survival curves showing overall survival (OS) of TCGA AML patients stratified by MALNC expression (CPM cut-off 0.5), P-value determined by the Mantel–Cox log-rank test. The sample number is indicated in parentheses (n).

Figure S9

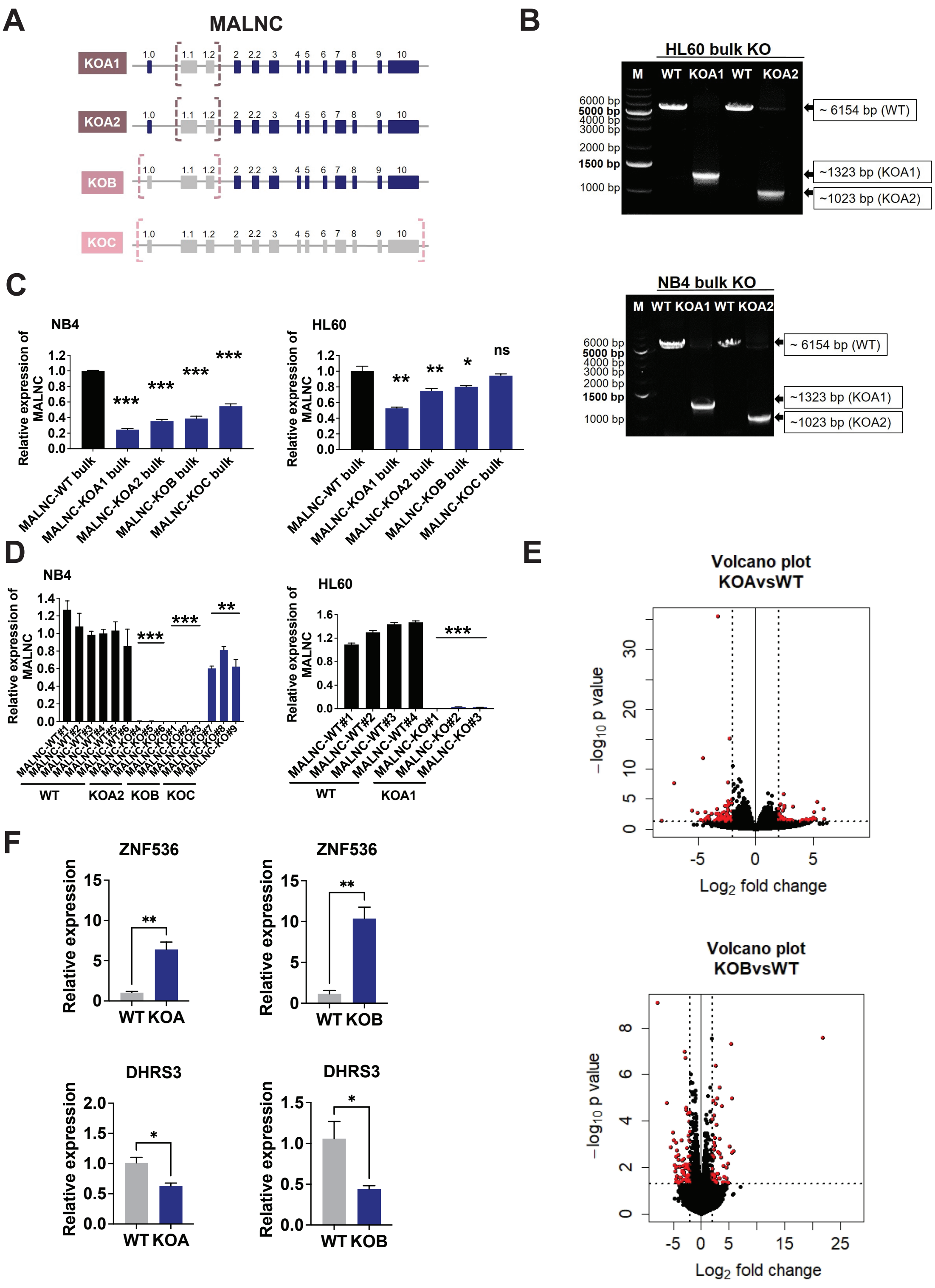
A
MALNC
KOA1
KOA2
KOB
KOC
1.0 1.1 1.2 2 2.2 3 4 5 6 7 8 9 10
B
HL60 bulk KO
M WT KOA1 WT KOA2
6000 bp
5000 bp
4000 bp
3000 bp
2000 bp
1500 bp
1000 bp
~ 6154 bp (WT)
~1323 bp (KOA1)
~1023 bp (KOA2)
NB4 bulk KO
M WT KOA1 WT KOA2
~ 6154 bp (WT)
~1323 bp (KOA1)
~1023 bp (KOA2)
C
NB4
HL60
Relative expression of MALNC
MALNC-WT bulk
MALNC-KOA1 bulk
MALNC-KOA2 bulk
MALNC-KOB bulk
MALNC-KOC bulk
ns
D
NB4
HL60
WT
KOA2
KOB
KOC
KOA1
E
Volcano plot
KOAvsWT
Volcano plot
KOBvsWT
−log10 p value
Log2 fold change
F
ZNF536
DHRS3
Relative expression
WT KOA
WT KOB

Fig. S9. *MALNC*^{KO} identifies genes associated with differentiation and retinoic acid signaling as MALNC-dependent. (A) Schematic overview depicting the four different CRISPR-generated KO versions of *MALNC* in HL60 and NB4 cells. KOA-1 and KOA-2 both delete two of three transcript starting exons (Exon 1.1 and Exon 1.2), removing about 5kb genomic sequence each. They differ by alternative usage of gRNA pairs. KOB version deletes all three starting exons (Exon 1.0, Exon 1.1 and Exon 1.2), removing about 16kb, and KOC deletes the whole transcript *loci* of *MALNC*, about 76kb. **(B)** Gel electrophoresis of PCR amplicons resulting from *MALNC*^{KO} on transfected bulk populations. Amplicons of 1323bp (KOA-1) and 1023bp (KOA-2), obtained both for HL60 and NB4, and amplicons of 915bp (KOB) and 842bp (KOC), obtained for NB4, indicate successful CRISPR deletion of *MALNC* on a bulk level. All amplicons were validated by Sanger sequencing. **(C)** Relative expression of MALNC (qRT-PCR) in *(right)* NB4 and *(left)* HL60 WT and KO bulk populations after CRISPR-RNP *MALNC*^{KO}. Data is shown as means ± SEM from three biological experiments relative to WT and normalized towards endogenous control gene *TBP*. **(D)** Relative expression of MALNC (qRT-PCR) in *(right)* NB4 and *(left)* HL60 WT and KO single cell clones after CRISPR-RNP *MALNC*^{KO} and single cell propagation. Data is shown as means ± SEM from three biological experiments relative to WT and normalized towards endogenous control gene *TBP*. **(E)** Volcano plots of differentially expressed genes between NB4-*MALNC KOA* and NB4-*MALNC*_WT and NB4-*MALNC KOB* and NB4-*MALNC*_WT. **(C)**Volcano plot of differentially expressed genes between NB4-*MALNC*$^{KOB\text{-}ATRA}$ and NB4-*MALNC*_WT$^{\text{-}ATRA}$. **(F)** Relative expression of ZNF536 and DHRS3 genes (qRT-PCR) in NB4-MALNC WT and NB4-*MALNC* KOA (left) and KOB (right) clones. Data is shown as means ± SEM from three biological experiments relative to WT and normalized towards endogenous control gene *TBP.* P-values were determined by Students t-tests: ns- not significant, *p-value < 0.05, **p-value < 0.01, ***p-value < 0.001, ****p-value < 0.0001.

Figure S10

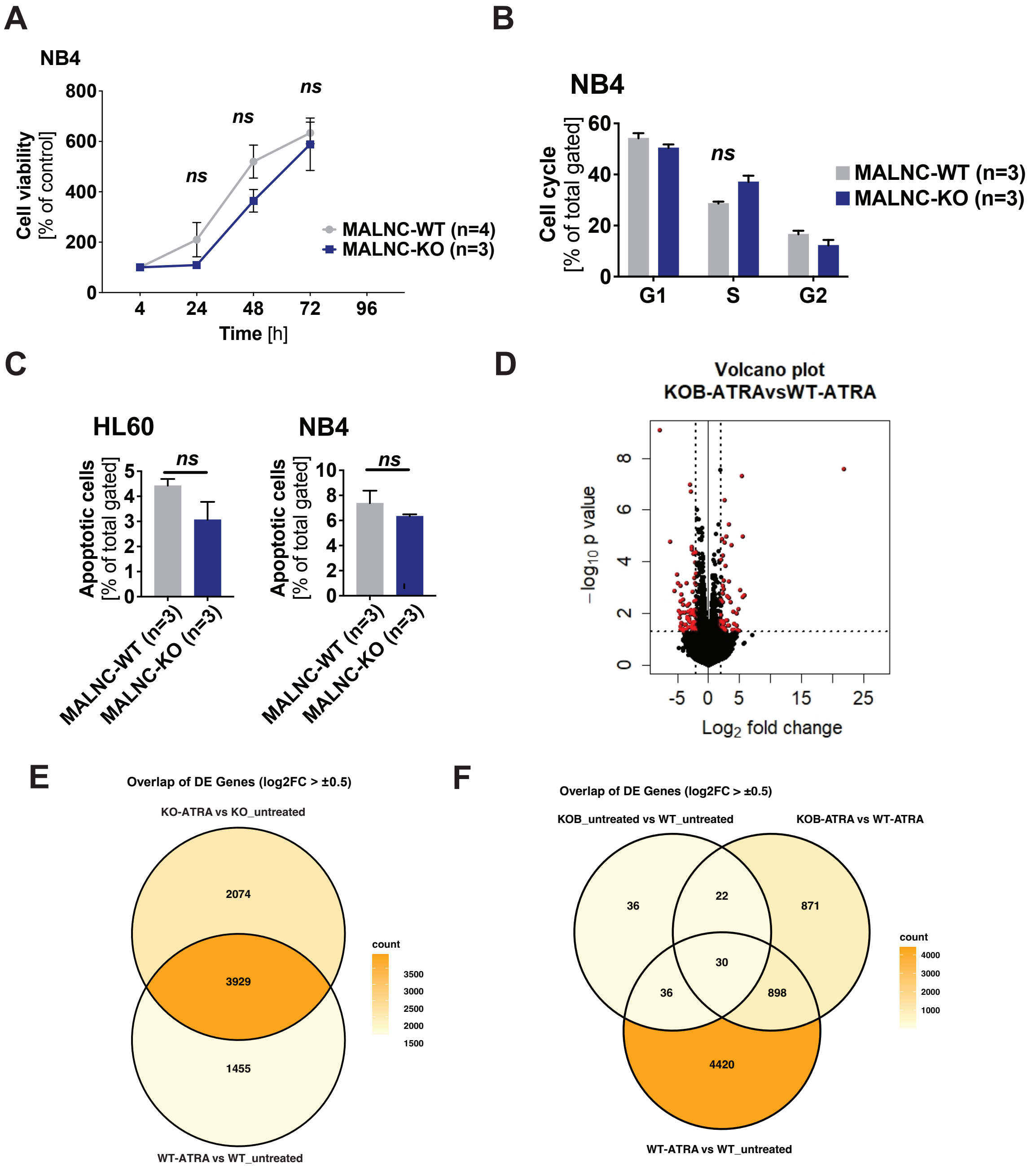
A
NB4
Cell viability [% of control]
Time [h]
ns
MALNC-WT (n=4)
MALNC-KO (n=3)
B
NB4
Cell cycle [% of total gated]
ns
G1
S
G2
MALNC-WT (n=3)
MALNC-KO (n=3)
C
HL60
NB4
Apoptotic cells [% of total gated]
ns
MALNC-WT (n=3)
MALNC-KO (n=3)
D
Volcano plot
KOB-ATRAvsWT-ATRA
−log10 p value
Log2 fold change
E
Overlap of DE Genes (log2FC > ±0.5)
KO-ATRA vs KO_untreated
WT-ATRA vs WT_untreated
2074
3929
1455
count
F
Overlap of DE Genes (log2FC > ±0.5)
KOB_untreated vs WT_untreated
KOB-ATRA vs WT-ATRA
WT-ATRA vs WT_untreated
36
22
871
30
36
898
4420
count

Fig. S10. ***MALNC***^{KO} **increases ATRA-induced myeloid differentiation.** **(A)** Cell viability in NB4 *MALNC*^{KO} (n=4), *MALNC*^{WT} (n=3) at the basal level. Cell viability in percent of control (4h) was measured by WST-8 reagent up to 72h. Data is shown as means ± SEM from three biological experiments**.** **(B)** Cell cycle analysis of NB4- *MALNC*^{KO} and NB4- *MALNC*^{WT} clones. Data was assessed by PI staining using flow cytometry after 72 H from cell seeding. Data is shown as means ± SEM from three biological experiments. **(C)** Apoptotic cells of HL60- and NB4-*MALNC*^{KO} (n=3) and *MALNC*^{WT} (n=3). Apoptosis is presented as a percent of gated cells 72h-post cell seeding. Data is shown as means ± SEM from three biological experiments. **(D)** Volcano plot of differentially expressed genes between NB4-*MALNC*$^{KOB-ATRA}$ and NB4-*MALNC*_WT^{-ATRA}. **(E)** Overlap of differentially expressed genes between NB4 *MALNC*-WT-ATRA vs NB4 *MALNC*-WT-untreated cells (n=5384) and NB4 *MALNC* KOB-ATRA vs NB4 *MALNC*-KOB-untreated cells (n=6003). DE genes were identified by DEseq2 using cut-off p-val adj < 0.05, log2FC |>0.5|. **(F)** Overlap of differentially expressed genes between NB4 *MALNC* KOB-untreated vs NB4 *MALNC* WT-untreated (n=124) with NB4 *MALNC* KOB-ATRA vs NB4 *MALNC* WT-ATRA (n=1821) and NB4 *MALNC* WT-ATRA vs NB4 *MALNC* WT untreated (n=5384) cells. DE genes were identified by DEseq2 using cut-off p-val adj < 0.05, log2FC |>0.5. P-values were determined by Students t-tests: ns- not significant, *p-value < 0.05, **p-value < 0.01, ***p-value < 0.001, ****p-value < 0.0001.

Figure S11

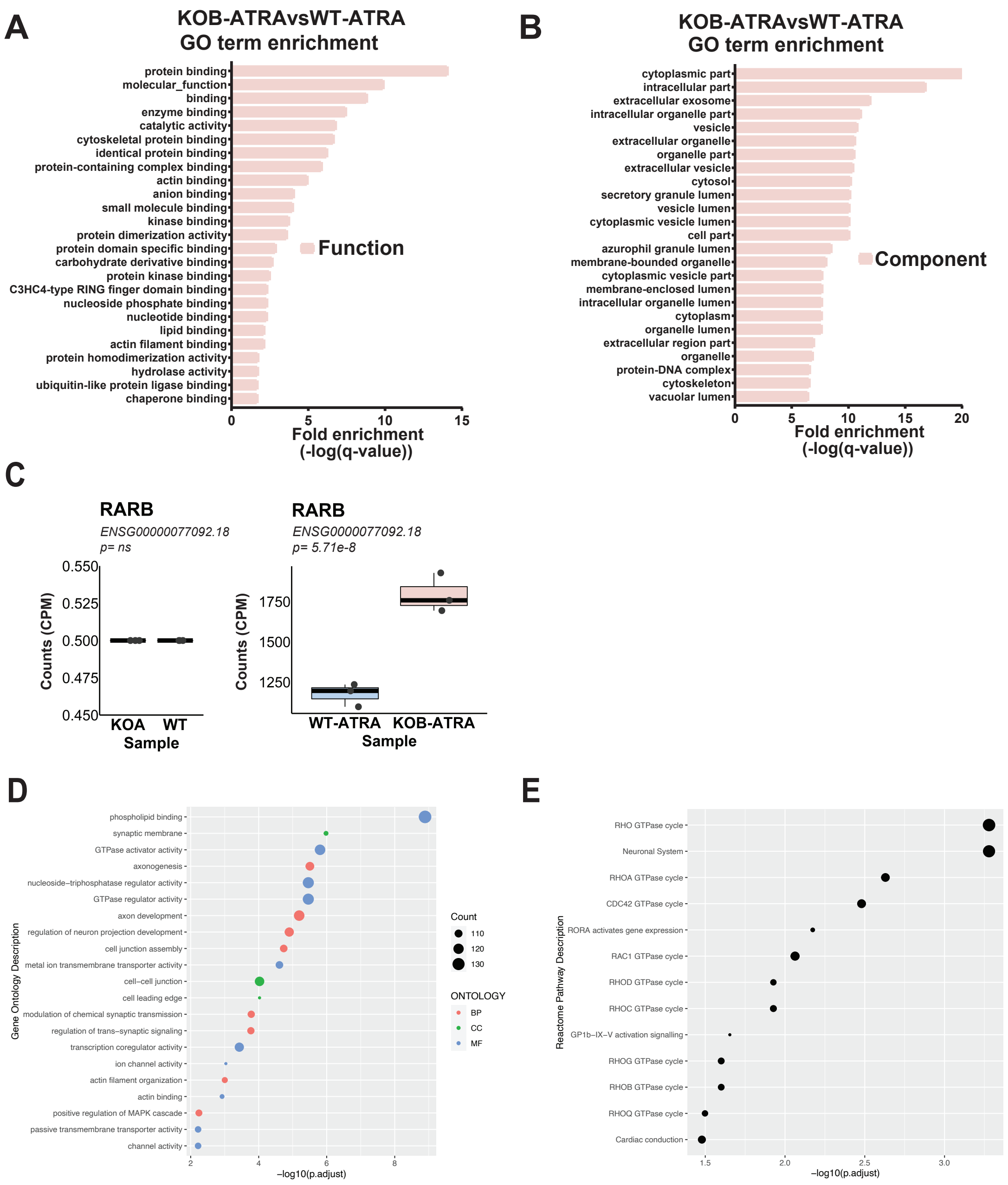
A
KOB-ATRAvsWT-ATRA
GO term enrichment
protein binding
molecular_function
binding
enzyme binding
catalytic activity
cytoskeletal protein binding
identical protein binding
protein-containing complex binding
actin binding
anion binding
small molecule binding
kinase binding
protein dimerization activity
protein domain specific binding
carbohydrate derivative binding
protein kinase binding
C3HC4-type RING finger domain binding
nucleoside phosphate binding
nucleotide binding
lipid binding
actin filament binding
protein homodimerization activity
hydrolase activity
ubiquitin-like protein ligase binding
chaperone binding
Function
0
5
10
15
Fold enrichment
(-log(q-value))
B
KOB-ATRAvsWT-ATRA
GO term enrichment
cytoplasmic part
intracellular part
extracellular exosome
intracellular organelle part
vesicle
extracellular organelle
organelle part
extracellular vesicle
cytosol
secretory granule lumen
vesicle lumen
cytoplasmic vesicle lumen
cell part
azurophil granule lumen
membrane-bounded organelle
cytoplasmic vesicle part
membrane-enclosed lumen
intracellular organelle lumen
cytoplasm
organelle lumen
extracellular region part
organelle
protein-DNA complex
cytoskeleton
vacuolar lumen
Component
0
5
10
15
20
Fold enrichment
(-log(q-value))
C
RARB
ENSG00000077092.18
p= ns
0.550
0.525
0.500
0.475
0.450
Counts (CPM)
KOA
WT
Sample
RARB
ENSG0000077092.18
p= 5.71e-8
1750
1500
1250
Counts (CPM)
WT-ATRA
KOB-ATRA
Sample
D
phospholipid binding
synaptic membrane
GTPase activator activity
axonogenesis
nucleoside−triphosphatase regulator activity
GTPase regulator activity
axon development
regulation of neuron projection development
cell junction assembly
metal ion transmembrane transporter activity
cell−cell junction
cell leading edge
modulation of chemical synaptic transmission
regulation of trans−synaptic signaling
transcription coregulator activity
ion channel activity
actin filament organization
actin binding
positive regulation of MAPK cascade
passive transmembrane transporter activity
channel activity
Gene Ontology Description
2
4
6
8
−log10(p.adjust)
Count
110
120
130
ONTOLOGY
BP
CC
MF
E
RHO GTPase cycle
Neuronal System
RHOA GTPase cycle
CDC42 GTPase cycle
RORA activates gene expression
RAC1 GTPase cycle
RHOD GTPase cycle
RHOC GTPase cycle
GP1b−IX−V activation signalling
RHOG GTPase cycle
RHOB GTPase cycle
RHOQ GTPase cycle
Cardiac conduction
Reactome Pathway Description
1.5
2.0
2.5
3.0
−log10(p.adjust)
Count
30
60
90

Fig. S11. ***MALNC binds to the chromatin of genes related to the Rho GTPase.*** **(A-B)** GO term enrichment analysis for function and GO term enrichment analysis for the component on differentially expressed genes dysregulated among KOB^{-ATRA} (ATRA treated NB4 *MALNC*^{KO}#2, KOB) and WT^{-ATRA} (ATRA-treated NB4 *MALNC*^{WT}#4). Gene ontology (GO) and pathway analysis were performed using GOrilla (Gene Ontology enRIchment anaLysis and visuaLizAtion tool). Analysis was performed using a target gene list against a background gene list with a q-value cut-off of 0.01. The target list consisted of DE genes (n=1821) between KOB^{-ATRA} (ATRA-treated NB4 *MALNC*^{KO}#2) versus WT^{-ATRA} (ATRA-treated NB4 *MALNC*^{WT}#4), KOB^{-ATRA}vsWT^{-ATRA}. Shown are the top 25 hits per GO term analysis. **(C)** Box and Whisker plots of *RARB* gene expression obtained from RNA-seq data from *MALNC*^{KO} and *MALNC*^{WT} comparison at basal level and under ATRA treatment conditions. **(D-E)** Gene ontology (*GO)* and Reactome pathway enrichment *analysis* of MALNC binding peaks identified through ChIRP-seq analysis. Genes are subdivided by GO and pathway enrichment analysis into Biological processes (BP), cellular components(CC) and molecular functions (MF). Gene count is represented with dot size.

.

References

1. Qu Y, Siggens L, Cordeddu L, Gaidzik VI, Karlsson K, Bullinger L, et al. Cancer-specific changes in DNA methylation reveal aberrant silencing and activation of enhancers in leukemia. Blood [Internet]. 2017;129(7):e13–25. Available from: http://dx.doi.org/10.1182/blood-2016-07-726877

2. Wang M, Lindberg J, Klevebring D, Nilsson C, Mer AS, Rantalainen M, et al. Validation of risk stratification models in acute myeloid leukemia using sequencing-based molecular profiling. Leukemia [Internet]. 2017/02/07. 2017 Oct;31(10):2029–36. Available from: https://pubmed.ncbi.nlm.nih.gov/28167833

3. Network CGAR, Ley TJ, Miller C, Ding L, Raphael BJ, Mungall AJ, et al. Genomic and epigenomic landscapes of adult de novo acute myeloid leukemia. N Engl J Med [Internet]. 2013/05/01. 2013 May 30;368(22):2059–74. Available from: https://pubmed.ncbi.nlm.nih.gov/23634996

4. Mujahed H, Miliara S, Neddermeyer A, Bengtzén S, Nilsson C, Deneberg S, et al. AML displays increased CTCF occupancy associated with aberrant gene expression and transcription factor binding. Blood [Internet]. 2020;136(3):339–52. Available from: http://dx.doi.org/10.1182/blood.2019002326

5. Tyner JW, Tognon CE, Bottomly D, Wilmot B, Kurtz SE, Savage SL, et al. Functional Genomic Landscape of Acute Myeloid Leukemia. Nature [Internet]. 2018 Oct 25 [cited 2024 Jan 9];562(7728):526. Available from: https://pubmed.ncbi.nlm.nih.gov/30333627

6. Anders S, Huber W. Differential expression analysis for sequence count data. Genome Biol [Internet]. 2010/10/27. 2010;11(10):R106–R106. Available from:

https://pubmed.ncbi.nlm.nih.gov/20979621

7. Love MI, Huber W, Anders S. Moderated estimation of fold change and dispersion for RNA-seq data with DESeq2. Genome Biol [Internet]. 2014;15(12):550. Available from: https://pubmed.ncbi.nlm.nih.gov/25516281

8. Eden E, Navon R, Steinfeld I, Lipson D, Yakhini Z. GOrilla: a tool for discovery and visualization of enriched GO terms in ranked gene lists. BMC Bioinformatics [Internet]. 2009 Feb 3;10:48. Available from: https://pubmed.ncbi.nlm.nih.gov/19192299

9. Pemovska T, Kontro M, Yadav B, Edgren H, Eldfors S, Szwajda A, et al. Individualized Systems Medicine Strategy to Tailor Treatments for Patients with Chemorefractory Acute Myeloid Leukemia. Cancer Discov [Internet]. 2013;3(12):1416–29. Available from: http://dx.doi.org/10.1158/2159-8290.cd-13-0350

10. Kang Y-J, Yang D-C, Kong L, Hou M, Meng Y-Q, Wei L, et al. CPC2: a fast and accurate coding potential calculator based on sequence intrinsic features. Nucleic Acids Res [Internet]. 2017 Jul 3;45(W1):W12–6. Available from: https://pubmed.ncbi.nlm.nih.gov/28521017

11. Wang L, Park HJ, Dasari S, Wang S, Kocher J-P, Li W. CPAT: Coding-Potential Assessment Tool using an alignment-free logistic regression model. Nucleic Acids Res [Internet]. 2013/01/17. 2013 Apr 1;41(6):e74–e74. Available from: https://pubmed.ncbi.nlm.nih.gov/23335781
